# The effect of Tabata-style functional high-intensity interval training on cardiometabolic health and physical activity in female university students

**DOI:** 10.3389/fphys.2023.1095315

**Published:** 2023-02-27

**Authors:** Yining Lu, Huw D. Wiltshire, Julien Steven Baker, Qiaojun Wang, Shanshan Ying

**Affiliations:** ^1^ Faculty of Sport Science, Ningbo University, Ningbo, China; ^2^ Cardiff School of Sport and Health Sciences, Cardiff Metropolitan University, Cardiff, United Kingdom; ^3^ Centre for Population Health and Medical Informatics, Department of Sport, Physical Education and Health, Hong Kong Baptist University, Kowloon Tong, Hong Kong SAR, China

**Keywords:** Tabata, functional training, cardiometabolic health, physical activity, young females

## Abstract

**Introduction:** The increasing prevalence of metabolic syndrome and physical inactivity enhances exposure to cardiometabolic risk factors in university students. High-intensity interval training (HIIT) improved cardiometabolic health in clinical adults but the evidence in the university setting is limited. Furthermore, few studies examined the effect of low-volume HIIT on habitual physical activity (PA). Therefore, the primary aim of this study was to evaluate the efficacy of 12-week Tabata-style functional HIIT for improving multiple cardiometabolic health outcomes and habitual PA. We also investigated whether changes in habitual PA over the intervention period had an impact on exercise-induced health outcomes.

**Methods:** 122 female freshmen were randomized into the Tabata group (n = 60) and the control (n = 62). The Tabata training protocol involved 8 × 20 s maximal repeated functional exercises followed by 10 s rest with a frequency of 3 times per week for 12 weeks. Body composition, maximal oxygen uptake (VO_2max_), blood pressure (BP), blood lipids, fasting glucose and insulin, C-reactive protein and PA were objectively measured using standardized methods. Dietary intake was measured using a valid food frequency questionnaire. All variables were measured pre- and post-intervention.

**Results:** Mixed linear modelling results showed that there were large intervention effects on VO_2max_ (*p* < 0.001, d = 2.53, 95% CI: 2.03 to 3.00 for relative VO_2max_; *p* < 0.001, d = 2.24, 95% CI: 1.76 to 2.68 for absolute VO_2max_), resting heart rate (*p* < 0.001, d = −1.82, 95% CI: −2.23 to −1.37), systolic BP (*p* < 0.001, d = −1.24, 95% CI: −1.63 to −0.84), moderate-to-vigorous intensity physical activity (MVPA) (*p* < 0.001, d = 2.31, 95% CI: 1.83 to 2.77), total PA (*p* < 0.001, d = 1.98, 95% CI: 1.53 to 2.41); moderate effects on %BF (*p* < 0.001, d = -1.15, 95% CI: −1.53 to −0.75), FM (*p* < 0.001, d = −1.08, 95% CI: −1.46 to −0.69), high-density lipoprotein (HDL) (*p* < 0.001, d = 1.04, 95% CI: 0.65 to 1.42), total cholesterol (*p* = 0.001, d = −0.64, 95% CI: −1.00 to −0.26); small effects on BMI (*p* = 0.011, d = −0.48, 95% CI: −0.84 to 0.11), WC (*p* = 0.043, d = −0.37, 95% CI: −0.74 to −0.01), low-density lipoprotein (*p* = 0.003, d = −0.57, 95% CI: −0.93 to −0.19), HOMA-IR (*p* = 0.026, d = −0.42, 95% CI: −0.78 to −0.05) and fasting insulin (*p* = 0.035, d = −0.40, 95% CI: −0.76 to −0.03). Regression analysis showed that only the percentage change of HDL was associated with the change of MVPA (b = 0.326, *p* = 0.015) and TPA (b = 0.480, *p* = 0.001).

**Conclusion:** From the findings of the study we can conclude that 12-week low-volume Tabata-style functional HIIT was highly effective for university female students to improve cardiorespiratory fitness, body fat, some cardiometabolic health outcomes and habitual PA.

## 1 Introduction

It is well acknowledged that the process of atherosclerosis begins in childhood, and with the accumulation of cardiometabolic risk factors, its clinical manifestations are often observed in late adulthood (Andersen et al., 2004; Oliveira et al., 2010). Even though cardiovascular disease (CVD) mortality has reduced sharply over the last few decades in middle-aged and elderly people, the reduction rate is lower in young adults (Yano, 2021). This may be due to the accelerated aggregation of cardiovascular risk factors, which results in the increasing prevalence of metabolic syndrome (MetS) in young adults, particularly in young women (Ford et al., 2004; Regitz-Zagrosek et al., 2007; Hirode and Wong, 2020). Substantial evidence shows that a healthy lifestyle, including maintaining a favorable body mass, engaging in high levels of leisure-time physical activity (PA), and healthy eating habits, play an important role against the risk of MetS [Dickie et al., 2014; Wu et al., 2016; Kass et al., 2017; Lv et al., 2017; Lim et al., 2021; ]. In addition, a high level of cardiorespiratory fitness (CRF) tends to have a protective effect against CVDs (Rankinen et al., 2007; Kim et al., 2014) and low levels of inflammation are associated with lower risks of future CVD events (Ridker et al., 2017; Zhu et al., 2018; Arnold et al., 2021). Unfortunately, a range of studies have found that these risk factors are not well controlled among university students, resulting in an increase of CVD risk in this population. For example, students are often observed to gain unhealthy body mass (Hovell et al., 1985; Vella-Zarb and Elgar, 2009), have low levels of leisure-time PA and prolonged sedentary time (Vella-Zarb and Elgar, 2009; Kwan et al., 2012; Kljajević et al., 2021), eat insufficient vegetables and fruits (El Ansari et al., 2012; Moreno-Gómez et al., 2012; Scarapicchia et al., 2015), and experience a downward trend in CRF (Scott et al., 2016; Lamoureux et al., 2019; Lan et al., 2022).

Although there have been multiple interventions aimed at improving university students’ lifestyles (Brown et al., 2014; Soriano-Ayala et al., 2020), PA levels (Sharp and Caperchione, 2016; Chiang et al., 2019; Heeren et al., 2018), or dietary habits (Lhakhang et al., 2014; Castillo et al., 2019; Whatnall et al., 2019; Hernández-Jaña et al., 2020), their effectiveness varied widely (Plotnikoff et al., 2015). This may be attributed to the absence of practice in most interventions. Practice is regarded as the key to the effectiveness of interventions, especially for those aimed at improving PA (Maselli et al., 2018). However, it seems to be a challenge to enforce a practical intervention in the university setting since lack of time is the most cited barrier to PA engagement among university students (Lovell et al., 2010), and participating in a practical trial will burden students further in addition to academic work (Sweeney, 2011). These provide a strong context for developing a novel intervention targeting modifiable risk factors associated with cardiovascular health in this population. To this end, a gender-specified strategy is necessary. Male and female students have different attitudes towards health promotion interventions (von Bothmer and Firdlund, 2005) and gender related differences in cardiovascular health risks can be observed. For example, women with insufficient PA are more likely to be obese (Vainshelboim et al., 2019). Particularly, obese young women are associated with increased risk of preterm delivery (Cnattingius et al., 2013), as well as impaired cognitive development of their infants (Casas et al., 2013). Given these findings, coupled with the low levels of PA (Haase et al., 2004; Grasdalsmoen et al., 2019) and the high prevalence of MetS and obesity in women (Riediger and Clara, 2011; Li et al., 2016; Ajlouni et al., 2020), an effective intervention to improve PA and cardiovascular health is warranted for female university students. Establishing an active lifestyle in early adulthood will contribute to long-term health (Jang and Kim, 2019).

High-intensity interval training (HIIT) has gained popularity among young people in recent years. HIIT is characterized by several short bouts of intermittent intense exercise interspersed with recovery periods of different durations (Laursen and Jenkins, 2002). Numerous data has shown that, compared to traditional moderate-intensity continuous training (MICT), HIIT can provide similar or greater improvements in maximal oxygen uptake (VO_2max_) with less exercise time and energy expenditure (Jelleyman et al., 2015; Milanović et al., 2015; Sultana et al., 2019). However, its effects on cardiometabolic health outcomes are controversial. The improvements in insulin sensitivity, blood pressure (BP), and body composition using HIIT were more likely to be observed in overweight or obese individuals, especially when they continued training for 12 weeks or longer (Kessler et al., 2012; Jelleyman et al., 2015; Batacan et al., 2017).

Several studies have evaluated the efficacy and feasibility of HIIT in university settings. Foster et al. (2015) evaluated the effect of 8-week Tabata training with eight intervals of 20-s cycling at 170% VO_2max_/10-s rest on VO_2max_ and reported a significant increase of 18% in VO_2max_ (Foster et al., 2015). Likewise, Eather et al. (2019) conducted an 8-week HIIT intervention using a frequency of three sessions per week. During each session, participants were required to complete a total of 8–12 min of training that included aerobic and strength exercises using the 30-s: 30-s work-rest ratio. After the intervention, CRF and muscular fitness were improved significantly whereas body composition had no gains (Eather et al., 2019). On the contrary, results from the study by Hu et al. (2022) showed that, after a 4-week functional exercise based HIIT, body composition, heart rate (HR), BP and arterial stiffness were improved in female university students with normal weight obesity (NWO). Although few improvements in cardiometabolic health were observed in short term HIIT (<12 weeks) (Batacan et al., 2017), the beneficial effects reported by Hu et al. (2022)’s study might be due in large part to the high levels of exercise intensity (90% of HR_max_), during (3 × 9 minutes/session) and frequency (5 sessions/week) selected. Coupled with the obese participants, it was not surprising to see several improvements in cardiometabolic outcomes H following Hu et al. (2022)’s study. However, the total workout time of about 30 min seemed to go against the time-efficient nature of HIIT; Zhang et al. (2017) compared the fat-reducing effect of HIIT (90% of VO_2max_) and MICT (60% of VO_2max_) in obese female university students. After the 12-week intervention, participants in the HIIT group had similar reductions in percentage body fat (%BF) and total fat mass (FM) as those in the MICT group. Although the HIIT group had significantly shorter exercise durations than the MICT group, lasting nearly 30 min (Zhang et al., 2017). Improvements on CRF were consistent after HIIT. A recent meta-analysis of randomized controlled trials reported that HIIT protocols with short-intervals (≤30 s), low-volume (≤5 min) and short-term (≤4 weeks) were all effective in increasing VO_2max_ (Wen et al., 2019). However, with respect to outcomes regarding to cardiometabolic health and body composition, the effects of HIIT appears to be more dependent on the FITT principle (frequency, intensity, times and type) as well as the baseline characteristics of participants.

Tabata training is recognized as one of the most efficient forms of HIIT. A Tabata-style HIIT protocol is characterized by its unique training procedure that comprises of eight bouts of 20-s exercise followed by a 10-s rest (Tabata, 2019). While the feasibility of the supramaximal intensity of 170% of VO_2max_ from its original protocol has been questioned (Gentil et al., 2016), studies have found health benefits when it is performed at an intensity of 70%–80% of HR_max_ (Menz et al., 2019; Popowczak et al., 2022). With a total of 4 min, the Tabata-style HIIT protocol had been reported to significantly improve both aerobic and anerobic capacities (Tabata et al., 1996; Murawska-Cialowicz et al., 2020). This was supported by a recent systematic review (Viana et al., 2019). Although results from this systematic review demonstrated limited evidence on the weight-reducing effect of the Tabata protocol, some improvements on body composition were reported in the study by Murawska-Cialowicz et al. (2020) and Domaradzki et al. (2020). In addition, BP (Popowczak et al., 2022), fat oxidation (Pearson et al., 2020) and muscular performance (Menz et al., 2019; Islam et al., 2020) benefited from Tabata-style HIIT using functional exercises.

Nevertheless, there is concern that high perceived exertion and low enjoyment are associated with future PA and exercise adherence, especially in participants with a low level of CRF (Dishman, 1994; Follador et al., 2018). Intense exercises appeared to induce a subsequent decline in non-exercise PA and an increase in sedentary time (Skovgaard et al., 2019; Joshi and Dodge, 2022). This is thought to be a compensatory behavior, where the energy expended during exercises needs to be compensated for in other behaviors (Skovgaard et al., 2019). The compensatory behaviors may explain in part why the intervention did not produce the results expected (King et al., 2008). The short-term effects on compensatory movement behaviors following a Tabata-style HIIT had been investigated in our previous study and the results showed an increase in both sedentary time and moderate-to-vigorous intensity physical activity (MVPA) (Lu et al., 2022a).

Therefore, the primary purpose of this study is to evaluate the effectiveness of a Tabata-style functional HIIT on PA and cardiometabolic health in female university students with assessments at baseline and after 12 weeks of supervised training. We hypothesize that Tabata training is effective in improving cardiometabolic health and PA. Meanwhile, within the intervention group, subgroup analysis is planned to explore whether there are differential effects between normal weight and overweight/obese participants. We hypothesize that overweight/obese participants had greater improvements compared to normal weight ones after intervention. We further examine whether changes in cardiometabolic outcomes after the intervention are associated with changes in PA. We expected to see a positive relationship between changes in cardiometabolic outcomes and changes in PA.

## 2 Methods

### 2.1 Participants

**FIGURE 1:**
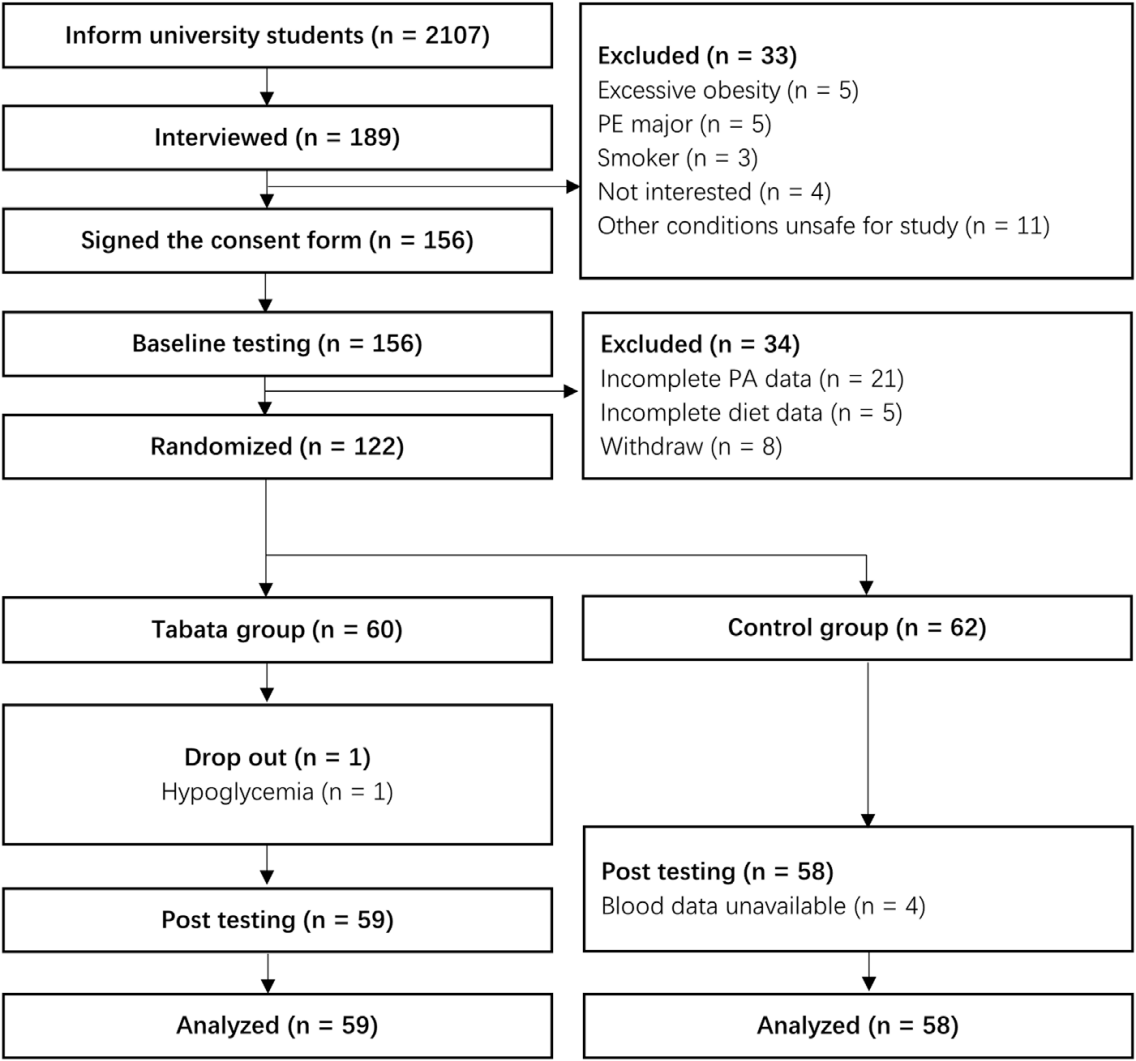
The process of sample and study timeline. Note: PA, physical activity; PE, physical education.

This study was approved by Ningbo University ethics committee on 23 February 2022 (RAGH20220166). The participants recruitment process began in March 2022. Female students who enrolled in September 2021 from Ningbo University were invited to participate in the study *via* mobile messages and WeChat groups. A presentation was conducted during weekly PE classes comprising of an introduction, practical demonstration, and question and answer session. The students who showed interest were then interviewed face-to-face. During the interview, participants who decided to take part in the study were instructed to sign an informed consent form and join a WeChat group. Students with symptoms of or diagnosed CVDs, diabetes, or had any other conditions that might affect PA and dietary intake were excluded. In addition, female students who were pregnant or had the likelihood of pregnancy were also excluded. Students who confirmed participation were required to complete all pre-intervention measurements by the end of March 2022. Finally, 122 female students who had complete PA, diet, laboratory, biochemical, and lifestyle data were enrolled in this study. The process of sample and study timeline were outlined in Figure 1.

### 2.2 Study design

The study was a 12-week randomized controlled trial examining the effectiveness of a Tabata-style functional HIIT on PA and cardiometabolic health in female university students. Participants were randomly assigned to either Tabata or control groups. A complete randomization was used, and the random assignment was conducted using the RAND function in Excel. A researcher recorded the details of eligible participants in excel and assigned excel-generated random numbers to each of them. The random numbers given to the participants were ranked from the smallest to the largest, and the first 60 participants were assigned to the intervention group and the second 62 to the control group. Given our limited number of accelerometers and HR monitors, each of which was only 30, and the total of 122 participants, we decided to allocate 60 participants to the intervention group. During the following exercise sessions, 30 participants worked out in a group. The participants were then informed *via* phone or message. Because this was a practical exercise intervention, blinding was useless for participants. However, researchers were blinded to group assignment during the post-intervention measurement and data analysis. Participants allocated to the Tabata or the control group were invited to join a separate WeChat group. Participants in the control group were instructed to keep their routine habits during the intervention period. Participants in the intervention group were required to complete a total of 36 sessions of a Tabata-style functional HIIT with the frequency of three sessions per week. Each session involved a total of 19 min exercises (10 min warm-up, 4 min Tabata workout, and 5 min cool-down). Training took place in an indoor gym and was supervised by researchers. Training times were allocated on Tuesdays, Thursdays and one of the weekends, with morning and afternoon sessions from 9 to 10 a.m. and 3 to 4 p.m., respectively. Participants chose the training session based on their time schedule and adhered to it throughout the intervention. If participants were unable to attend a scheduled session, they had to make it up the next day and be monitored by a researcher. Despite the intensity of training, the retention of participants was high, with only one participant dropping out due to the incidence of hypoglycemia during the first session. The study yielded a final analysis of 59 participants in the Tabata group. Baseline measurements were completed prior to the beginning of the 12-week intervention program and the post-training tests were conducted within the week immediately following the cessation of program. It should be noted that, due to the shortage of accelerometers, the post PA data were measured in groups. The post PA measure was conducted at intervention week 11 and 12 in the intervention group, and at the following 2 weeks in the control group. All measurements, except blood related ones, were completed in the laboratory of Research Academy of Grand Health from Ningbo University. Venous blood samples were collected from a superficial antecubital vein by qualified and experienced phlebotomists according to standard phlebotomy procedures in the affiliated hospital of medical school from Ningbo University. Participants were required to abstain from foods, drinks other than water, and strenuous exercises for at least 8 h before the blood measurements.

### 2.3 Physical, physiological and body composition measurements

Height was measured in duplicate, using a standard stadiometer protocol. Weight, %BF, FM, fat free mass (FFM) and basal metabolic rate (BMR) were measured using bioelectrical impedance analysis (MC-180, TANITA CO., China). Participants were required to empty their bladder to minimize measurement error caused by “electrically silent” (Kushner et al., 1996). Participants wearing normal PE clothing without any metal items were instructed to stand barefoot on the bioelectrical impedance analysis by trained researchers. Outcomes were obtained from the associated software. Body mass index (BMI) was calculated using standardized equations. Waist circumference (WC) was measured with a flexible steel tape to the nearest 0.1 cm. For WC, two measurements were taken and if the difference between two measurements was larger than 1%, a third measurement was needed. The mean value was used in the analysis if two measurements were taken, and the median value was used if three measurements were taken. The WHO recommended a BMI of 25 kg/m^2^ or higher as the cut-off point for overweight or obesity. Although this cut-off point is controversial for the Asian population, there is not enough evidence to indicate a clear cut-off point for Asians. Furthermore, BMI was not considered as a good predictor of CVDs and mortality. According to the optimal cut-off points for identification of the CVD risks in Chinese adults (Zhou, 2002; Yang et al., 2016), participants were classified as overweight or obesity if they met one of the following criteria: 1) BMI ≥24 kg/m^2^; 2) WC ≥ 80 cm; and 3) %BF ≥ 35%.

Resting HR (HR_resting_), systolic BP (SBP) and diastolic BP (DBP) were measured by trained researchers using an automatic upper arm sphygmomanometer (HEM-1000, Omron, China). Prior to BP measurement, participants were required to sit and rest for at least 5 min. BP measurements were taken in a seated position from the left arm, with the upper section of the arm supported at the heart level. Three measurements were performed at 1-min intervals and the average of the second and the third readings were used for analysis. If the two readings differed by more than 5 mm Hg, an additional measurement was taken. BP was classified based on the recommendation from the American Heart Association as normal (SBP <120 mm Hg and DBP <80 mm Hg), elevated (SBP = 120–129 mm Hg and DBP <80 mm Hg), hypertension (HTN) stage 1 (SBP = 130–139 mm Hg or DBP = 80–89 mm Hg), and HTN stage 2 (SBP ≥140 mm Hg or DBP ≥90 mm Hg). Participants with elevated, HTN stage 1 or stage 2 were identified as having unhealthy BP.

### 2.4 Cardiorespiratory fitness

VO_2max_ was used to measure the cardiorespiratory fitness of participants. The modified YMCA submaximal cycle ergometer test was used. Details of the VO_2max_ measurement has been provided in previous studies (Lu et al., 2022a).

### 2.5 Dietary intake

Dietary intake was assessed by a staff-administered semi-quantitative food frequency questionnaire (FFQ). This 63-item FFQ was modified from the validated questionnaire used in the 2015 China Nutrition and Health Survey (Zhao et al., 2002). The frequency and quantity of food intake in the past 12 months were estimated. The dietary intake was divided into nine categories: staple food, beans, vegetables, fruits, milk, meats, eggs, snacks, and alcohol and beverages. The consumption frequency included: 1) never, 2) times per year, 3) times per month, 4) times per week, and 5) times per day and participants need to answer only one of these questions. The consumption amount for each time was recorded as Gram or ml. Samples were presented to help participants more accurately record serving amount. The intake of nutrients was calculated according to the Chinese Food Composition Tables (Yang YX and Pan, 2002) and manufacturer information.

### 2.6 Physical activity

Details of PA measurements had been provided in the previous study (Lu et al., 2022a). In brief, PA was measured using a triaxial accelerometer (ActiGraph, wGT3X-BT, Pensacola, FL, United States). Participants were instructed to wear the accelerometer on the non-dominant hip for seven consecutive days except during water-based activities. A valid day was defined as not less than 75% of the wear time between 7 a.m. and 11 p.m. and participants provided at least four valid days including at least one weekend were included in the final analysis. The intensity of PA was classified according to the Freedson Adult algorithm. Sedentary was defined as < 100 counts per minute (cpm), light intensity physical activity (LPA) was defined as 100–1951 cpm, moderate intensity physical activity (MPA) was defined as 1952–5,724 cpm, vigorous intensity physical activity (VPA) as > 5,725 cpm, and MVPA as > 1952 cpm. Total physical activity (TPA) was defined as the daily vector magnitude cpm.

### 2.7 Cardiometabolic measurements

Generally, it was recommended to use fasting blood samples for blood profiling. In contrast, most of the 24 h were in a non-fasting state and the non-fasted lipid profile was suggested to better capture atherogenic lipoprotein levels (Nordestgaard, 2017). Moreover, the non-fasting blood collection would help recruit and retain participants. However, considering the large sample size and the flexibility of blood collecting time, we decided to use fasting blood to control measurement bias. Fasting blood samples were collected into EDTA-treated vacutainers and analyzed using standardized procedures in the hospital laboratory (Power Processor, Beckman Coulter’s complete range of clinical lab automation systems, United States). Samples will be analyzed for lipids (total cholesterol (TC), low-density lipoprotein (LDL), high-density lipoprotein (HDL), triglycerides (TG), HbA1c, C-reactive protein (CRP), fasting glucose (FPG) and fasting insulin. HOMA-IR was calculated (from measures described above) as follows: fasting insulin (μU/ml) x fasting glucose (mmol/L)/22.5. MetS was defined as having 3 or more of the following five abnormalities: 1) central obesity (WC: women ≥80 cm); 2) elevated TG (≥150 mg/dL (≥1.7 mmol/L)) or drug treatment; 3) low HDL (women <50 mg/dL (<1.3 mmol/L)) or drug treatment; 4) HTN (SBP ≥130 and/or DBP ≥85 mm Hg) or drug treatment; 5) elevated FPG (>100 mg/dL (>5.6 mmol/L)) or drug treatment (Alberti et al., 2009).

### 2.8 Interventions

Prior to the first session, participants were instructed to perform a familiarization session, during which 4 functional movements (jumping jacks, high knees, squat jumps, and mountain climbers in sequence) and their sequence of exercise was recorded. The researchers used the “Timer Plus” App to keep time and verbally count down 3 s to the end of each workout and rest. During the exercise, a chest strap HR monitor (Polar H10, Polar, Malaysia) was used to record HR data per second. Monitors were placed near the heart and attached by a band to the chest using non-slip silicone dots and a buckle, which did not make participants uncomfortable and affect exercise performance. HR data was processed using the Polar Flow. In this familiarization session, age-predicted HR_max_ (220—age) was used to examine the exercise intensity. According to the Tabata protocol, 90% of HR_max_ was required during the sixth bout. For example, a 20-year-old participant had to reach a 180 beats per minute (bpm) of HR. After the exercise, participants would be informed whether they performed at the target intensity. The 90% cut-off point was only used for researchers as the criterion for satisfactory delivery of Tabata training. Participants received qualitative feedbacks such as “you should go faster” or “you did a good job”. This feedback enabled them to perceive and familiarize themselves with the intensity required during the training. However, the age predicted HR_max_ was reported to overestimate among young adults (Gellish et al., 2007). Since all participants were freshmen with few age differences, the use of age-predicted HR_max_ equations appeared to result in excessive intensity for participants, especially for those with lower fitness. Therefore, during the formal intervention, the intensity of 90% HR_max_ was not compulsive. Participants would receive the feedback about their performance after each exercise session.

All exercise sessions began with a 10-min low-to-moderate warm-up. The warm-up exercises involved joints movement, static stretching, and dynamic stretching. The HR was required to reach 60% of HR_max_ during the warm-up. In the 4-min Tabata training, four functional movements were performed using participants’ own body weight. These movements were selected based on the Tabata training recommendations (Tabata, 2019) and showed a good acceptance in female university students in the polit study (Lu et al., 2022a). Participants were encouraged to repeat the movement as many times as possible during the 20-s workout, then rest for 10 s. The four movements were performed in sequence and then repeated, with a total of 4 min exercise. There was a 5-min cool down and stretching after the 4-min workout.

### 2.9 Exercise fidelity

Participants were instructed to wear a HR monitor (Polar H10, Polar, Malaysia), which was able to record HR data at 1-s intervals. The HR_mean_ and HR_peak_ of each session were recorded and presented as a percentage of the individual’s HR_max_. Participants’ HR_max_ was estimated using the conventional age-predicted equation used during the familiarization session. Although this equation was limited in predicting the accurate HR_max_, with a high variability of 12 bpm among subjects of identical age, it was still recommended in clinical settings and published in resources by well-established organizations in the field (Fletcher et al., 2013). Furthermore, since the individualized exercise prescription was not the primary objective of the present study, the utility of age predicted HR_max_ appeared to be acceptable and reasonable. In our previous study, the HR_mean_ achieved during exercise was 82.4% ± 1.9% of age predicted HR_max_ (Lu et al., 2022a). To reflect the high intensity, a cut-point of 80% of HR_max_ was used to evaluate the intervention fidelity (Garber et al., 2011). Participants with a HR_mean_ below the 80% of HR_max_ over the 12-week intervention were excluded in the final analysis.

### 2.10 Other variables

Other variables including demographic data, lifestyle, and family history of HTN and type 2 diabetes were identified using a standardized questionnaire. Smoking, drinking alcohol, and staying up late were classified as never, sometimes, or always. The family history of HTN and type 2 diabetes were classified as yes or no.

### 2.11 Statistical analysis

All statistical analyses were performed using IBM SPSS for windows, version 23.0 (Chicago, IL, United States) and the significance level was set as *p* < 0.05.

Sample size was estimated by G * Power (version 3.1.9.7) (Heinrich Heine University, Dusseldorf, Germany) using *a priori* in relation to the primary outcome for this study. According to our previous study (Lu et al., 2022a), we calculated a correlation between groups of 0.70 and the effect size of 0.28 for the sample size estimation. The power and alpha were set at 0.95 and 0.05, respectively. Using a t-tests marched pairs design, 20 participants were required for the Tabata group.

Data normality was examined using the Kolmogorov-Smirnov and Shapiro-Wilk tests. Logarithms were used for non-normality data. In the descriptive statistical analysis, participants were categorized into groups based on obesity status. Descriptive analyses were summarized as means with 95% confidence intervals (CI), and proportions for continuous and categorical variables, respectively. For variables that were logarithmically transformed, geometric means and geometric standard deviations were used. ANOVA and χ2 test were used to analyze differences between continuous and categorical variables, respectively. Paired *t*-test was used to explore any differences between pre- and post-intervention within groups. Correlations between variables were examined using the Pearson product moment correlation coefficient. Mixed linear models were used to evaluate intervention effects between the Tabata and the control group, considering the percentage change in the measurements between the posttest and pretest as outcomes. Cohen’s d was used to provide a measure of effect size (mean difference on percentage change [posttest − pretest] between the Tabata and the control group over the intervention divided by the pooled SD of percentage change). The effect size was classified as trivial (<0.2), small (0.2–0.6), moderate (0.6–1.2), or large (>1.2) (Hopkins et al., 2009). Mixed linear models were also used to explore the moderating effect of weight status (normal weight vs overweight/obese) with interaction terms (intervention × weight status). The same statistical methods were used for subgroup analyses.

For cardiometabolic risk factors with significant changes after intervention, linear regression models were used to test the association between changes in cardiometabolic outcomes and changes in PA (MVPA and TPA). The variance inflation factor (VIF) was used to check the multicollinearity and an issue of multicollinearity was identified if VIF >5.0. Due to the high correlation between the change of MVPA and the change of TPA, for each cardiometabolic outcome, two models were tested. The dependent variable was the percentage change of cardiometabolic outcome. The independent variable was the percentage change of MVPA in model 1, and the percentage change of TPA in model 2. All models controlled for age, lifestyle variables, the baseline, and the percentage change of body composition, CRF and dietary variables, the baseline value of PA data, as well as the baseline value of the cardiometabolic outcome modelled. Participants’ ID numbers were used for data store and processing. Participants with less than 90% attendance were excluded in the final analysis (Hernández-Jaña et al., 2020).

## 3 Results

### 3.1 Descriptive statistics

The baseline characteristics of participants are presented in Table 1. One participant in the Tabata group dropped out the intervention due to the incidence of hypoglycemia. Four participants in the control group missed the post-test blood data. Therefore, a total of 59 participants in the Tabata group and 58 participants in the control were included in the final analysis.

**TABLE 1:** Baseline characteristics of participants.

Variables	All (n = 117)	Tabata (n = 59)	Control (n = 58)	*p*-value
Age (years)	20.38 (20.14 to 20.63)	20.42 (20.03 to 20.82)	20.34 (20.03 to 20.65)	*p* > 0.05
Height (cm)	163.48 (162.61 to 164.35)	163.88 (162.70 to 165.07)	163.07 (161.77 to 164.37)	*p* > 0.05
Alcohol				*p* > 0.05
Never	108 (92.31%)	55 (93.22%)	53 (91.38%)	
Sometimes	9 (7.69%)	4 (6.78%)	5 (8.62%)	
Always				
Staying up late				*p* > 0.05
Never				
Sometimes	42 (35.90%)	20 (33.90%)	22 (37.93%)	
Always	75 (64.10%)	39 (66.10%)	36 (62.07%)	
Family history of hypertension				*p* > 0.05
Yes	6 (5.13%)	3 (5.08%)	3 (5.17%)	
No	111 (94.87%)	56 (94.92%)	55 (94.83%)	
Family history of diabetes				*p* > 0.05
Yes	3 (2.56%)	2 (3.39%)	1 (1.72%)	
No	114 (97.44%)	57 (96.61%)	57 (98.28%)	
Weight (kg)	56.60 (55.19 to 58.01)	56.23 (54.13 to 58.33)	56.97 (55.03 to 58.92)	*p* > 0.05
BMI (kg/m^2^)	21.16 (20.69 to 21.63)	20.92 (20.22 to 21.63)	21.40 (20.76 to 22.04)	*p* > 0.05
WC (cm)	73.82 (72.91 to 74.72)	73.92 (72.59 to 75.24)	73.72 (72.45 to 74.98)	*p* > 0.05
%Body fat	27.42 (26.69 to 28.14)	27.94 (26.79 to 29.08)	26.88 (25.97–27.80)	*p* > 0.05
FM (kg)	15.71 (14.98–16.43)	15.90 (14.80–16.99)	15.52 (14.54–16.49)	*p* > 0.05
FFM (kg)	40.89 (40.07–41.72)	40.34 (39.06–41.61)	41.46 (40.39–42.52)	*p* > 0.05
Basal Energy expenditure (kcal)	1234.33 (1219.07–1249.60)	1236.17 (1212.74–1259.60)	1232.47 (1212.24–1252.70)	*p* > 0.05
VO2max (mL/kg/min)	34.45 (33.72–35.19)	34.24 (33.21–35.27)	34.67 (33.59–35.76)	*p* > 0.05
VO2max (L/min)	1.94 (1.89–2.00)	1.92 (1.84–1.99)	1.97 (1.88–2.06)	*p* > 0.05
Resting Heart Rate (bpm)	89.48 (87.76–91.20)	89.03 (86.55–91.52)	89.93 (87.47–92.39)	*p* > 0.05
SBP (mm Hg)	122.19 (120.03–124.29)	121.37 (118.37–124.38)	123.02 (120.00–126.04)	*p* > 0.05
DBP (mm Hg)	70.43 (69.29–71.56)	71.46 (69.88–73.04)	69.38 (67.74–71.01)	*p* > 0.05
HDL (mmol/L)	1.75 (1.65–1.84)	1.72 (1.58–1.85)	1.78 (1.65–1.91)	*p* > 0.05
LDL (mmol/L)	2.58 (2.48–2.69)	2.63 (2.47–2.78)	2.54 (2.40–2.68)	*p* > 0.05
TG (mmol/L)	1.10 (1.01–1.18)	1.09 (0.97–1.22)	1.10 (0.98–1.21)	*p* > 0.05
TC (mmol/L)	4.94 (4.81–5.08)	4.91 (4.70–5.11)	4.98 (4.80–5.16)	*p* > 0.05
HbA1c (%)	5.08 (5.03–5.14)	5.06 (4.99–5.14)	5.11 (5.03–5.18)	*p* > 0.05
FPG (mmol/L)	4.92 (4.83–5.01)	4.93 (4.81–5.04)	4.91 (4.76–5.05)	*p* > 0.05
Fasting insulin (μU/mL)	4.58 (4.38–4.79)	4.62 (4.34–4.90)	4.55 (4.24–4.85)	*p* > 0.05
HOMA-IR	1.00 (0.95–1.05)	1.01 (0.95–1.07)	0.99 (0.92–1.07)	*p* > 0.05
CRP (mg/L)	0.81 (0.79–0.83)	0.79 (0.76–0.82)	0.82 (0.80–0.85)	*p* > 0.05
MVPA (min/d)	103.68 (100.66–106.70)	105.14 (100.72–109.56)	102.20 (97.98–106.41)	*p* > 0.05
TPA (cpm)	1189.26 (1142.38–1236.13)	1167.21 (1099.78–1234.63)	1211.69 (1145.04–1278.33)	*p* > 0.05
Energy intake (kcal/day)	1752.56 (1661.31–1843.82)	1706.27 (1591.21–1821.32)	1799.66 (1655.01–1944.30)	*p* > 0.05
Vegetable (g/d)	263.85 (241.97–285.72)	249.90 (220.29–279.51)	278.04 (245.30–310.78)	*p* > 0.05
Fruit (g/d)	202.83 (181.28–224.37)	217.59 (183.39–251.79)	187.81 (161.27–214.34)	*p* > 0.05

Note: BMI, body mass index; BMR, basal metabolic rate; CRP, C-reactive protein; cpm, count per minute; DBP, diastolic blood pressure; FFM, fat-free mass; FM, fat mass; FPG, fasting glucose; HbA1c, glycosylated hemoglobin; HDL, high-density lipoprotein; HOMA-IR, homeostasis model assessment of insulin resistance; LDL, low-density lipoprotein; MVPA, moderate-to-vigorou intensity physical activity; SBP, systolic blood pressure; TC, total-cholesterol; TG, triglyceride; TPA, total physical activity; VO_2max_, maximal oxygen uptake; WC, waist circumference.

At baseline, according to the definition of overweight and obesity used in this study, 27 (23.08%) participants were classified as overweight or obese. Based on the MetS definition used in this study, the prevalence of MetS was 5.13%. For individual components of MetS, high BP was the most prevalent, with 36 (30.77%) participants had a SBP ≥130 mm Hg. 25 (21.37%) participants had low HDL, 20 (17.09%) participants had central obesity, 13 (11.11%) had elevated TG, and 10 (8.55%) had elevated FPG. 43 (36.75%) participants had one MetS component, 22 (18.80%) participants had two MetS components and 46 (39.32%) participants had none of these. Participants showed a relatively high level of PA, with only one participant failed to meet the minimum level of MVPA recommended. The average daily PA was 103.68 (95% CI: 100.66 to 106.70) minutes for MVPA and 1189.26 (95% CI: 1142.38 to 1236.13) cpm for TPA. Based on the recommended daily vegetable (300–500 g) and fruit (200–350 g) intake for Chinese people (Gu et al., 2021), most participants failed to meet the minimum daily vegetable and fruit intake (64.96% for vegetable and 53.85% for fruit). The average daily vegetable and fruit intake were 263.85 (95% CI: 241.97 to 285.72) g and 202.83 (95%CI: 181.28 to 224.37) g, respectively. Participants consumed an average of 1752.56 (95%CI: 1661.31 to 1843.82) calories per day. Alcohol and smoking were less common among female university students, with only 15 participants reporting alcohol drinking sometimes and no participants reporting smoking. Family history of hypertension and diabetes were reported by 6 and 3 participants, respectively. Staying up late was more common among participants, with 27 participants reporting staying up late sometimes and 90 reporting always. The relative and absolute cardiorespiratory fitness (VO_2max_) was 34.45 (95% CI: 33.72 to 35.19) mL/kg/min and 1.94 (95% CI: 1.89 to 2.00) L/min, respectively. According to the CRF percentiles recommended by Kaminsky et al. (2017), 24 (20.51%) participants were below the 50th percentile VO_2max_ of women aged 20 to 29 years of 31.0 mL/kg/min. Table 1 presented the separate data for Tabata and control groups and there were no significant between-group differences in parameters between groups at baseline.

### 3.2 Exercise fidelity

The average HR_max_ for the total Tabata group was 199.58 ± 1.52 bpm. All participants met the minimum level of intensity and a total of 2,124 individual heart rate data were analyzed. Over the 12-week intervention, the HR_mean_ during exercise was 83.24% (95% CI: 82.75% to 83.74%) of individual HR_max_, with a between-subject SD of 1.89%. The HR_mean_ varied between participants from 80.8% to 86.6%. The within-subject SD was 0.24%. The participants’ HR_mean_ varied from 82.77% to 83.77% over 36 exercise sessions. The HR_peak_ for exercise session across the intervention was 93.32% (95% CI: 92.52% to 94.11%) of individual HR_max_, with a between-subject SD of 3.05%. The HR_peak_ varied between participants from 89.8% to 97.3%. The within-subject SD was 0.30%. The participants’ HR_peak_ varied from 92.84% to 93.91% within different exercise sessions over the intervention. There were no differences between HR_mean_ and HR_peak_ during session 1 to session 12.

### 3.3 Post-intervention effects

The total time spent over the 12-week intervention was 684 min, with 360 min for warming up, 144 min for Tabata training, and 180 min for cooling down and stretching. Over the intervention, participants performed a total of 96-min of high-intensity exercise (8 min per week). Only 1 participant dropped out the intervention, resulting in a satisfied exercise adherence of 98.31%. The effects of the Tabata style functional HIIT are presented in Table 2. There were significant (*p* < 0.05) interaction effects for WC (*p* = 0.002), MVPA (*p* = 0.007), TPA (*p* = 0.009), LDL (*p* = 0.004), TG (*p* = 0.010), TC (*p* < 0.001), daily energy intake (*p* = 0.012) and vegetable intake (*p* = 0.001). Therefore, within the Tabata group, we also conducted a separate analysis for participants with overweight or obesity and those with normal weight (Table 3).

**TABLE 2:** The effects of the Tabata style functional HIIT between Tabata and control groups.

	Tabata (n = 59)			Control (n = 58)			Difference between groups		
	Preintervention mean (95% CI)	Postintervention mean (95% CI)	Mean change (95% CI)	Preintervention mean (95% CI)	Postintervention mean (95% CI)	Mean change (95% CI)	Mean change (95% CI)	*p*-value	Cohen’s d (95% CI)
Weight (kg)	56.23 (54.13–58.33)	56.04 (54.24–57.85)	-0.06% (-0.71% to 0.58%)	56.97 (55.03–58.92)	57.60** (55.74–59.46)	1.25% (0.46% to 2.03%)	-1.31% (-2.31% to -0.31%)	0.011	-0.48 (-0.84 to -0.11)
BMI (kg/m^2^)	20.92 (20.22–21.63)	20.86 (20.25–21.47)	-0.06% (-0.71% to 0.58%)	21.40 (20.76–22.04)	21.64** (21.03–22.25)	1.25 (0.46% to 2.03%)	-1.31% (-2.31% to -0.31%)	0.011	-0.48 (-0.84 to -0.11)
WC (cm)	73.92 (72.59–75.24)	73.78 (72.54–75.02)	-0.16 (-0.34% to 0.02%)	73.72 (72.45–74.98)	73.77 (72.53–75.01)	0.08 (-0.07% to 0.24%)	-0.24% (-0.48% to -0.01%)	0.043	-0.37 (-0.74 to -0.01)
%Body fat	27.94 (26.79–29.08)	27.15*** (26.08–28.21)	-2.57% (-4.06% to -1.09%)	26.88 (25.97–27.80)	27.69*** (26.83–28.55)	3.23% (2.10% to 4.36%)	-5.80% (-7.65% to -3.95%)	< 0.001	-1.15 (-1.53 to -0.75)
FM (kg)	15.90 (14.80–16.99)	15.34*** (14.40–16.27)	-2.61% (-4.36% to -0.85%)	15.52 (14.53–16.49)	16.12*** (15.18–17.06)	4.58% (2.86% to 6.30%)	-7.19% (-9.62% to 4.76%)	< 0.001	-1.08 (-1.46 to -0.69)
FFM (kg)	40.34 (39.06–41.61)	40.71* (39.53–41.88)	1.07% (0.37% to 1.77%)	41.46 (40.39–42.52)	41.48 (40.45–42.51)	0.13% (-0.51% to 0.77%)	0.94% (-0.00% to 1.88%)	0.051	0.37 (0.00–0.73)
Basal metabolic rate (kcal)	1236 (1213–1260)	1247** (1226–1268)	0.95% (0.33% to 1.56%)	1232 (1212–1253)	1235 (1215–1254)	0.20% (-0.24% to 0.64%)	0.75% (-0.00% to 1.50%)	0.050	0.37 (0.00–0.73)
VO2max (mL/kg/min)	34.24 (33.21–35.27)	38.56*** (37.43–39.69)	12.87% (11.00% to 14.73%)	34.67 (33.59–35.76)	34.57 (33.51–35.63)	-0.27% (-0.66% to 0.13%)	13.13% (11.23% to 15.04%)	< 0.001	2.53 (2.03–3.00)
VO2max (L/min)	1.92 (1.84–1.99)	2.15*** (2.07–2.24)	12.73% (11.02% to 14.44%)	1.97 (1.88–2.06)	1.99 (1.90–2.07)	0.98% (0.08% to 1.88%)	11.75% (9.82% to 13.67%)	< 0.001	2.24 (1.76–2.68)
Resting Heart Rate (bpm)	89.03 (86.55–91.52)	80.98*** (79.17–82.80)	-8.62% (-10.34% to -6.89%)	89.93 (87.47–92.39)	89.97 (87.55–92.38)	0.05% (-0.27% to 0.37%)	-8.66% (-10.42% to -6.92%-)	< 0.001	-1.82 (-2.23 to -1.37)
SBP (mm Hg)	121.37 (118.37–124.38)	116.56*** (114.63–118.49)	-3.66% (-4.73% to -2.58%)	123.02 (119.99–126.04)	123.28 (120.27–126.28)	0.22% (-0.20% to 0.64%)	-3.88% (-5.02% to -2.73%)	< 0.001	-1.24 (-1.63 to -0.84)
DBP (mm Hg)	71.46 (69.88–73.04)	71.44 (70.01–72.87)	0.06% (-0.41% to 0.54%)	69.38 (67.74–71.01)	69.48 (67.92–71.05)	0.19% (-0.09% to 0.47%)	-0.13% (-0.68% to 0.42%)	0.641	-0.09 (-0.45 to 0.28)
Lipid profile									
HDL (mmol/L)	1.72 (1.58–1.85)	1.82*** (1.71–1.94)	8.54% (5.82% to 11.27%)	1.78 (1.65–1.91)	1.78 (1.65–1.91)	0.45% (-0.40% to 1.30%)	8.09% (5.25% to 10.94%)	< 0.001	1.04 (0.65–1.42)
LDL (mmol/L)	2.63 (2.47–2.78)	2.56* (2.42–2.71)	-1.95% (-3.53% to -0.38%)	2.54 (2.40–2.68)	2.56 (2.43–2.69)	1.06% (-0.11% to 2.23%)	-3.01% (-4.96% to -1.07%)	0.003	-0.57 (-0.93 to -0.19)
TG (mmol/L)	1.09 (0.97–1.22)	1.06** (0.94–1.18)	-1.08% (-2.85% to 0.69%)	1.10 (0.98–1.21)	1.10 (0.99–1.21)	0.83% (-0.18% to 1.83%)	-1.91% (-3.93% to 0.12%)	0.065	-0.34 (-0.71 to 0.02)
TC (mmol/L)	4.91 (4.70–5.11)	4.83* (4.67–5.00)	-1.07% (-2.00% to -0.13%)	4.98 (4.80–5.16)	5.02** (4.85–5.19)	0.90% (0.25% to 1.55%)	-1.97% (-3.10% to -0.84%)	0.001	-0.64 (-1.00 to -0.26)
Carbohydrate metabolism									
HbA1c (%)	5.06 (4.99–5.14)	5.06 (4.98–5.13)	-0.17% (-0.36% to 0.02%)	5.11 (5.03–5.18)	5.10 (5.03–5.17)	-0.04% (-0.16% to 0.09%)	-0.13% (-0.36% to 0.09%)	0.241	-0.23 (-0.59 to 0.14)
FPG (mmol/L)	4.93 (4.81–5.04)	4.90 (4.80–4.99)	-0.43% (-1.09% to 0.23%)	4.91 (4.76–5.05)	4.90 (4.76–5.04)	-0.05% (-0.30% to 0.20%)	-0.38% (-1.08% to 0.33%)	0.291	-0.20 (-0.56 to 0.17)
HOMA-IR	1.01 (0.95–1.07)	0.99** (0.94–1.05)	-1.24% (-2.50% to 0.03%)	0.99 (0.92–1.07)	1.00 (0.93–1.08)	0.24% (-0.07% to 0.54%)	-1.47% (-2.76% to -0.18%)	0.026	-0.42 (-0.78 to -0.05)
Endocrine regulators									
Fasting insulin (μU/mL)	4.62 (4.34–4.90)	4.56* (4.31–4.80)	-0.83% (-1.82% to 0.17%)	4.55 (4.24–4.85)	4.56 (4.25–4.86)	0.29% (0.00% to 0.57%)	-1.11% (-2.15% to -0.08%)	0.035	-0.40 (-0.76 to -0.03)
Inflammation									
CRP (mg/L)	0.79 (0.76–0.82)	0.80 (0.77–0.83)	0.46% (-0.03% to 0.96%)	0.82 (0.80–0.85)	0.83 (0.80–0.85)	0.45% (-0.03% to 0.93%)	0.01% (-0.67% to 0.70%)	0.971	0.01 (-0.36–0.37)
Physical activity									
MVPA (min/d)	105.14 (100.72–109.56)	121.08*** (116.02–126.13)	15.51% (13.27% to 17.74%)	102.20 (97.98–106.41)	98.52** (95.10–101.95)	-2.95% (-4.88% to -1.03%)	18.46% (15.54% to 21.38%)	< 0.001	2.31 (1.83–2.77)
TPA (cpm)	1167.21 (1099.78–1234.63)	1327.48*** (1263.52–1391.44)	14.92% (12.33% to 17.51%)	1211.69 (1145.04–1278.33)	1176.20*** (1121.21–1231.19)	-2.03% (-3.85% to -0.22%)	16.95% (13.81% to 20.09%)	< 0.001	1.98 (1.53–2.41)
Dietary intake									
Energy intake (kcal/day)	1706.27 (1591.21–1821.32)	1694.23 (1601.69–1786.77)	0.85% (-1.36% to 3.06%)	1799.66 (1655.01–1944.30)	1806.37 (1663.25–1949.48)	0.60% (-0.19% to 1.39%)	0.26% (-2.08% to 2.59%)	0.829	0.04 (-0.32–0.40)
Vegetable (g/d)	249.90 (220.29–279.51)	249.40 (221.12–277.69)	0.90% (-0.46% to 2.27%)	278.04 (245.30–310.78)	277.24 (244.81–309.68)	0.01% (-0.60% to 0.63%)	0.89% (-0.60% to 2.39%)	0.238	0.22 (-0.15–0.58)
Fruit (g/d)	217.59 (183.39–251.79)	216.09 (182.84–249.34)	1.34% (-2.00% to 4.68%)	187.81 (161.27–214.34)	187.12 (160.67–213.57)	0.02% (-0.85% to 0.88%)	1.33% (-2.11% to 4.77%)	0.446	0.14 (-0.22–0.50)

Note: BMI, body mass index; BMR, basal metabolic rate; CRP, C-reactive protein; cpm, count per minute; DBP, diastolic blood pressure; FFM, fat-free mass; FM, fat mass; FPG, fasting glucose; HbA1c, glycosylated hemoglobin; HDL, high-density lipoprotein; HOMA-IR, homeostasis model assessment of insulin resistance; LDL, low-density lipoprotein; MVPA, moderate-to-vigorou intensity physical activity; SBP, systolic blood pressure; TC, total-cholesterol; TG, triglyceride; TPA, total physical activity; VO2max, maximal oxygen uptake; WC, waist circumference; *, difference between pre- and post-test, *p* < 0.05; **, *p* < 0.01; ***, *p* < 0.001.

**TABLE 3:** The effects of the Tabata style functional HIIT between overweight/obese and normal weight groups.

	Overweight/Obese (n = 10)			Normal weight (n = 49)			Adjusted difference between groups		
	Preintervention mean (95% CI)	Postintervention mean (95% CI)	Mean change (95% CI)	Preintervention mean (95% CI)	Postintervention mean (95% CI)	Mean change (95% CI)	Mean change (95% CI)	*p*-value	Cohen’s d
									(95% CI)
Weight (kg)	69.09 (64.67–73.51)	66.62*** (62.51–70.73)	-3.54% (-4.63% to -2.45%)	53.61 (52.04–55.18)	53.89 (52.48–55.29)	0.65% (0.08% to 1.21%)	-4.18% (-5.51% to -2.86%)	< 0.001	-2.19
									(-2.95 to -1.38)
BMI (kg/m2)	25.21 (24.29–26.14)	24.31*** (23.50–25.12)	-3.54% (-4.63% to -2.45%)	20.05 (19.47–20.62)	20.16 (19.63–20.68)	0.65% (0.08% to 1.21%)	-4.18% (-5.51% to -2.86%)	< 0.001	-2.19
									(-2.95 to -1.38)
WC (cm)	82.66 (81.38–83.94)	81.63* (80.31–82.95)	-1.24% (-2.13% to -0.35%)	72.13 (71.16–73.11)	72.18** (71.21–73.14)	0.06% (0.02% to 0.10%)	-1.30% (-1.66% to -0.95%)	< 0.001	-2.56 (-3.35 to -1.71)
%Body fat	32.79 (29.58–36.00)	30.99** (28.13–33.85)	-5.28% (-8.04% to -2.52%)	26.95 (25.89–28.01)	26.36** (25.31–27.41)	-2.02% (-3.71% to -0.33%)	-3.26% (-7.16% to 0.64%)	0.100	-0.58 (-1.26 to 0.12)
FM (kg)	22.67 (20.02–25.31)	20.66*** (18.30–23.03)	-8.61% (-11.85% to -5.37%)	14.51 (13.73–15.30)	14.25* (13.53–14.97)	-1.38% (-3.24% to 0.48%)	-7.23% (-11.54% to -2.93%)	0.001	-1.17 (-1.86 to -0.44)
FFM (kg)	46.43 (42.91–49.94)	45.96 (42.75–49.17)	-0.91% (-2.31% to 0.49%)	39.10 (37.98–40.21)	39.64*** (38.57–40.70)	1.47% (0.71% to 2.23%)	-2.37% (-4.15% to -0.60%)	0.010	-0.93 (-1.62 to -0.22)
Basal metabolic rate (kcal)	1334 (1247–1420)	1331 (1246–1415)	-0.19% (-0.84% to 0.45)	1216 (1197–1236)	1230** (1213–1247)	1.18% (0.46%–1.90)	-1.37% (-2.99% to 0.24%)	0.094	-0.59 (-1.27 to 0.10)
VO2max (mL/kg/min)	31.73 (28.88–34.58)	37.11*** (33.52–40.70)	16.98% (12.68% to 21.28%)	34.75 (33.66–35.84)	38.85*** (37.65–40.06)	12.03% (9.98% to 14.08%)	4.96% (0.11% to 9.80%)	0.045	0.71 (0.01–1.39)
VO2max (L/min)	2.20 (1.93–2.47)	2.47*** (2.18–2.77)	12.82% (8.90% to 16.74%)	1.86 (1.79–1.93)	2.09*** (2.01–2.17)	12.71% (10.75% to 14.67%)	0.11% (-4.50% to 4.71%)	0.963	0.02 (-0.66–0.70)
Resting Heart Rate (bpm)	91 (83–98)	82* (76–88)	-9.13% (-15.94% to -2.31%)	89 (86–91)	81*** (79–83)	-8.51% (-10.23% to -6.80%)	-0.61% (-5.24% to 4.02%)	0.792	-0.09 (-0.77 to 0.59)
SBP (mm Hg)	121 (115–127)	117* (113–121)	-3.35% (-6.12% to -0.57%)	121 (118–125)	117*** (114–119)	-3.72% (-4.92% to −2.51%)	0.37% (−2.52% to 3.26%)	0.798	0.09 (−0.59–0.77)
DBP (mm Hg)	71 (68–74)	71 (68–73)	0.07% (−1.22% to 1.36%)	72 (70–73)	72 (70–73)	0.06% (−0.47% to 0.59%)	0.01% (−1.27% to 1.28%)	0.991	0.01 (−0.67–0.69)
Lipid profile									
HDL (mmol/L)	1.69 (1.28–2.11)	1.88*** (1.51–2.26)	13.65% (6.73% to 20.57%)	1.72 (1.58–1.87)	1.81*** (1.69–1.93)	7.50% (4.52% to 10.48%)	6.15% (−0.99% to 13.28%)	0.090	0.60 (−0.10–1.28)
LDL (mmol/L)	3.07 (2.74–3.41)	2.81* (2.43–3.18)	-8.59% (−15.09% to −2.10%)	2.53 (2.37–2.70)	2.51 (2.36–2.67)	-0.60% (−1.81% to 0.61%)	-8.00% (−11.67% to 4.32%)	< 0.001	-1.51 (−2.22 to −0.76)
TG (mmol/L)	1.57 (1.41–1.73)	1.47** (1.33–1.61)	-6.30% (−9.23% to −3.37%)	1.00 (0.86–1.13)	0.98 (0.85–1.11)	-0.02% (−1.96% to 1.93%)	-6.28% (−10.75% to −1.82%)	0.007	-0.98 (−1.67 to −0.26)
TC (mmol/L)	5.67 (5.27–6.06)	5.38** (5.14–5.61)	-4.83% (−7.31% to −2.35%)	4.75 (4.54–4.96)	4.72 (4.54–4.91)	-0.30% (−1.20% to 0.60%)	-4.53% (−6.74% to −2.31%)	< 0.001	-1.42 (−2.12 to −0.67)
Carbohydrate metabolism									
HbA1c (%)	5.39 (5.28–5.51)	5.35 (5.26–5.45)	-0.73% (−1.48% to 0.03%)	5.00 (4.92–5.08)	4.99 (4.92–5.07)	-0.06% (−0.23% to 0.12%)	-0.67% (−1.15% to −0.19%)	0.008	-0.97 (−1.66 to −0.25)
FPG (mmol/L)	4.91 (4.58–5.23)	4.83* (4.56–5.10)	-1.50% (−2.92% to −0.09%)	4.93 (4.80–5.06)	4.91 (4.81–5.02)	-0.21% (−0.95% to 0.54%)	-1.30% (−3.04% to 0.45%)	0.142	-0.52 (−1.19 to 0.18)
HOMA-IR	1.21 (1.04–1.38)	1.16** (1.02–1.30)	-3.84% (−6.11% to −1.57%)	0.97 (0.90–1.04)	0.96 (0.90–1.01)	-0.70% (−2.13% to 0.72)	-3.13% (−6.42% to 0.15%)	0.061	-0.66 (−1.34 to 0.04)
Endocrine regulators									
Fasting insulin (μU/mL)	5.58 (4.73–6.42)	5.42* (4.70–6.14)	-2.36% (−4.52% to −0.19%)	4.43 (4.15–4.70)	4.38 (4.14–4.62)	-0.51% (−1.64% to 0.61%)	-1.84% (−4.48% to 0.79%)	0.166	-0.49 (−1.17 to 0.20)
Inflammation									
CRP (mg/L)	0.92 (0.87–0.96)	0.92 (0.88–0.97)	0.36% (−0.49% to 1.21%)	0.77 (0.73–0.80)	0.77 (0.74–0.80)	0.49% (−0.10% to 1.07%)	-0.13% (−1.46% to 1.20%)	0.848	-0.07 (−0.75 to 0.61)
Physical activity									
MVPA (min/d)	102.22 (86.04–118.41)	124.80*** (105.30–144.30)	22.50% (17.76% to 27.24%)	105.74 (101.20–110.27)	120.32*** (115.24–125.39)	14.08% (11.71% to 16.45%)	8.42% (2.85% to 14.00%)	0.004	1.05 (0.33–1.74)
TPA (cpm)	1178.83 (1047.84–1309.81)	1439.70*** (1307.90–1571.49)	22.84% (17.07% to 28.60%)	1164.84 (1086.40–1243.27)	1304.58*** (1232.33–1376.83)	13.30% (10.56% to 16.04%)	9.54% (3.05% to 16.02%)	0.005	1.02 (0.30–1.71)
Dietary intake									
Energy intake (kcal/day)	2,148.36 (1872.15–2,424.56)	2008.19* (1789.30–2,227.08)	-5.78% (−11.31% to −0.24%)	1616.05 (1501.67–1730.42)	1630.16 (1534.86–1725.46)	2.21% (−0.11% to 4.52%)	-7.98% (−13.54% to −2.42%)	0.006	-1.00 (−1.69 to −0.28)
Vegetable (g/d)	211.42 (135.71–287.13)	220.30* (145.48–295.12)	5.37% (0.49% to 10.25%)	257.75 (224.85–290.66)	255.34 (223.92–286.77)	-0.01% (−1.28% to 1.26%)	5.38% (1.99% to 8.77%)	0.002	1.10 (0.38–1.80)
Fruit (g/d)	115.71 (79.04–152.37)	117.59 (81.37–153.81)	2.28% (−0.92% to 5.48%)	238.38 (200.15–276.61)	236.19* (199.01–273.37)	1.15% (−2.85% to 5.16%)	1.13% (−7.85% to 10.11%)	0.803	0.09 (−0.59–0.77)

Differences between groups were adjusted for training groups (morning or afternoon).

Note: BMI, body mass index; BMR, basal metabolic rate; CRP, C-reactive protein; cpm, count per minute; DBP, diastolic blood pressure; FFM, fat-free mass; FM, fat mass; FPG, fasting glucose; HbA1c, glycosylated hemoglobin; HDL, high-density lipoprotein; HOMA-IR, homeostasis model assessment of insulin resistance; LDL, low-density lipoprotein; MVPA, moderate-to-vigorou intensity physical activity; SBP, systolic blood pressure; TC, total-cholesterol; TG, triglyceride; TPA, total physical activity; VO2max, maximal oxygen uptake; WC, waist circumference; *, difference between pre- and post-test, *p* < 0.05; **, *p* < 0.01; ***, *p* < 0.001.

### 3.4 Body composition

Body mass and BMI were unchanged in the Tabata group after intervention, while they increased in the control group (1.25%, 95% CI: 0.46% to 2.03%), with statistically significant differences on percentage changes between Tabata group and control group (*p* = 0.011, d = −0.48, 95% CI: −0.84 to −0.11). Both effects were small in magnitude. There was no statistically significant change in WC for both groups while a statistically significant differences on percentage changes was observed (*p* = 0.043, d = −0.37, 95% CI: −0.74 to −0.01). The intervention effect was small on WC. There were significant decreases in %BF (−2.57%, 95%CI: −4.06% to −1.09%) and FM (−2.61%, 95% CI: −4.36% to −0.85%) for the Tabata group, with moderate intervention effects between the Tabata group and control group (*p* < 0.001, d = −1.15, 95% CI: −1.53 to −0.75 for %BF; *p* < 0.001, d = −1.08, 95% CI: −1.46 to −0.69 for FM). FFM and metabolic basal rate (MBR) were improved significantly in the Tabata group (1.07%, 95% CI: 0.37% to 1.77% for FFM; 0.95%, 95% CI: 0.33% to 1.56% for MBR) and no significant intervention effects were observed between two groups (*p* = 0.051 and *p* = 0.050 for FFM and BMR, respectively).

Subgroup analysis showed that weight and BMI significantly decreased in participants with overweight or obesity only (−3.54%, 95% CI: −4.63% to −2.45%). There were significant differences on percentage changes on weight and BMI between the overweight/obese group and the normal weight group after adjusting for the training group (morning/afternoon group). (*p* < 0.001, d = −2.19, 95% CI: −2.95 to −1.38). The effects were large in magnitude. Although WC was not improved after intervention in the pooled sample in Tabata group, a significant decrease on WC was observed in participants with overweight or obesity (−1.24%, 95% CI: −2.13% to −0.35%). Even though %BF and FM decreased significantly in both normal weight and elevated weight participants; we observed significant differences on percentage changes on FM between groups (*p* = 0.001, d = −1.17, 95% CI: −1.86 to −0.44). Among overweight or obesity participants, we observed a decrease of 5.28% (95% CI: 2.52% to 8.04%) for %BF and 8.61% (95% CI: 5.37% to 11.85%) for FM. There was a decline of 2.02% (95% CI: −0.33% to −3.71%) for %BF and 1.38% (95% CI: −0.48% to 3.24%) for FM in normal-weight participants. Only participants with normal weight had an increase in FFM and RBR after intervention (1.47%, 95% CI: 0.71% to 2.23%). There was a significant difference on percentage change on FFM between groups (*p* = 0.010, d = −0.93, 95% CI: −1.62 to −0.22).

### 3.5 Cardiorespiratory fitness

After the 12-week intervention, both relative and absolute VO_2max_ were significantly improved in the Tabata group (12.87%, 95% CI: 11.00% to 14.73% for relative VO_2max_; 12.73%, 95% CI: 11.02% to 14.44% for absolute VO_2max_), with a large intervention effect between groups (*p* < 0.001, d = 2.53, 95% CI: 2.03 to 3.00 for relative VO_2max_; *p* < 0.001, d = 2.24, 95% CI: 1.76 to 2.68 for absolute VO_2max_). Neither relative nor absolute VO_2max_ increased in the control. We found a significant decrease in HR_resting_ in Tabata group only (−8.62%, 95% CI: −10.34% to −6.89%), with a significant between-group difference (*p* < 0.001, d = −1.82, 95% CI: −2.23 to −1.37). The intervention effect was large.

Subgroup analysis showed that there was a significant difference on percentage change on relative VO2max between the overweight/obese participants and the normal weight counterparts (*p* = 0.045, d = 0.71, 95% CI: 0.01 to 1.39). The effect was moderate. After the intervention, participants with overweight or obesity had a greater improvement in relative VO_2max_ of 16.98% (95% CI: 12.68% to 21.28%) compared with those with normal weight (12.03%, 95% CI: 9.98% to 14.08%). However, no between-group differences were observed on absolute VO_2max_ (*p* = 0.963) or HR_resting_ (*p* = 0.792).

### 3.6 Blood pressure

For BP data, only SBP decreased significantly in the Tabata group (−3.66%, 95% CI: −4.73% to −2.58%), with a large intervention effect (*p* < 0.001, d = −1.24, 95% CI: −1.63 to −0.84). DBP was unchanged after intervention in both groups. Among the Tabata group, there was no difference on percentage change on SBP (*p* = 0.798) or DBP (*p* = 0.991) between participants with overweight or obesity and those with normal weight.

### 3.7 Lipid profiles

HDL significantly increased in the Tabata group (8.54%, 95% CI: 5.82% to 11.27%), with a moderate intervention effect between groups (*p* < 0.001, d = 1.04, 95% CI: 0.65% to 1.42%). Tabata group showed significant decreases in LDL (−1.95%, 95% CI: −3.53% to −0.38%), TG (−1.08%, 95% CI: −2.85% to 0.69%) and TC (−1.07%, 95% CI: −2.00% to −0.13%), whereas control group demonstrated no changes in LDL and TG and a significant increase in TC (0.90%, 95% CI: 0.25% to 1.55%). A small intervention effect on LDL (*p* = 0.003, d = -0.57, 95% CI: −0.93 to -0.19) and a moderate effect on TC (*p* = 0.001, d = −0.64, 95% CI: −1.00 to −0.26) were observed.

Subgroup analysis showed significant differences in LDL (*p* < 0.001, d = −1.51, 95% CI: −2.22 to −0.76), TG (*p* = 0.007, d = −0.98, 95% CI: −1.67 to −0.26) and TC (*p* < 0.001, d = −1.42, 95%CI: −2.12 to -0.67) between overweight/obese participants and their normal weight counterparts. The effects were large in LDL and TC, and the effect was moderate in TG. Significant improvements in LDL, TG and TC were only observed in overweight/obese participants9, with a decrease of 8.59% (95% CI: 2.10% to 15.09%), 6.30% (95% CI: 3.37% to 9.23%) and 4.83% (95% CI: 2.35% to 7.31%) for LDL, TG and TC, respectively. Both overweight/obese participants (13.65%, 95% CI: 6.73% to 20.57%, *p* < 0.001) and their normal weight counterparts had a significant improvement in HDL (7.50%, 95% CI: 4.52% to 10.48%, *p* < 0.001). However, there was no statistically significant difference on the percentage change on HDL between groups (*p* = 0.090).

### 3.8 Carbohydrate metabolism and endocrine regulators

There were no significant changes in FPG and HbA1c for both groups, with no intervention effect. HOMA-IR improved significantly in the Tabata group (−1.24%, 95% CI: −2.50% to 0.03%) after intervention. There was a small intervention effect on HOMA-IR between groups (*p* = 0.026, d = −0.42, 95% CI: −0.78 to −0.05). Similarly, a small intervention effect was observed on fasting insulin (*p* = 0.035, d = −0.40, 95% CI: −0.76 to −0.03). There was a 0.83% (95% CI: −0.17% to 1.82%) decrease in fasting insulin from preintervention to postintervention for those in the Tabata group (*p* = 0.018).

Although FPG was not improved in the overall sample in the Tabata group, in the subgroup analysis it significantly decreased in overweight or obese participants (−1.50%, 95% CI: −2.92% to −0.09%, *p* = 0.034). There was a significant difference in percentage change in HbA1c between the overweight/obese group and the normal weight group (*p* = 0.008, d = −0.97, 95% CI: −1.66 to −0.25). The effect was moderate. HOMA-IR and fasting insulin were improved only in the overweight or obesity group (−3.84%, 95% CI: −6.11% to −1.57%, *p* = 0.009 for HOMA-IR; −2.36%, 95% CI: −4.52% to −0.19%, *p* = 0.042), while no effects on weight status was observed (*p* = 0.061 for HOMR-IR; *p* = 0.166 for fasting insulin).

### 3.9 Inflammation markers

There was no statistically significant change in CRP for both groups after the intervention, with no statistically significant intervention effect (*p* = 0.971). Subgroup analysis showed no significant improvement in CRP in either the overweight/obese or the normal weight group.

### 3.10 Metabolic syndrome

Following 12-week intervention, the prevalence of MetS decreased significantly in the Tabata group from 5.08% to 0.00%, whereas it did not change significantly in the control group (5.17% vs 5.17%). Among the Tabata group, 27 (79.41%) participants improved at least 1 component of MetS after training. Among the remaining seven participants, 2 of them had insufficient improvement of SBP, two participants had insufficient improvement of WC, 1 participant had insufficient improvement of TG and 1 participant had insufficient improvement of HDL. For the remaining 1 participant, both WC and HDL were not improved sufficiently to below cut points.

### 3.11 Physical activity

MVPA and TPA significantly increased in the Tabata group (15.51%, 95% CI: 13.27% to 17.74%, *p* < 0.001 for MVPA; 14.92%, 95% CI: 12.23% to 17.51% for TPA), whereas both MVPA and TPA decreased in the control group (−2.95%, 95% CI: −4.88% to −1.03%, *p* = 0.001 for MVPA; −2.03%, 95% CI: −3.85% to 0.22%, *p* < 0.001 for TPA), with a large intervention effect between groups (*p* < 0.001, d = 2.31, 95% CI: 1.83 to 2.77).

Subgroup analysis showed that there were significant differences in percentage change on MVPA (*p* = 0.004, d = 1.05, 95% CI: 0.33 to 1.74) and TPA (*p* = 0.005, d = 1.02, 95% CI: 0.30 to 1.71) between overweight/obese and normal weight groups after the intervention. Both effects were moderate. MVPA significantly increased by 22.50% (95% CI: 17.76% to 27.24%) and 14.08% (95% CI: 11.71% to 16.45%) for participants with overweight/obesity and normal weight, respectively (*p* < 0.001). TPA significantly increased by 22.84% (95% CI: 17.07% to 28.60%) and 13.30% (95% CI: 10.56% to 16.04%) for participants with overweight/obesity and normal weight, respectively (*p* < 0.001).

### 3.12 Dietary data

After intervention, both the Tabata group and control group showed slightly but not significant increases in dietary intake including energy intake, vegetable, and fruit intake. There were no intervention effects. While in the sub-group analysis, overweight/obese participants significantly decreased daily energy intake by 5.78% (95% CI: 0.24% to 11.31%) and increased vegetable intake by 5.37% (95% CI: 0.49% to 10.25%). There were significant differences on daily energy intake (*p* = 0.006, d = −1.00, 95% CI: −1.69 to −0.28) and vegetable intake (*p* = 0.002, d = 1.10, 95% CI: 0.38 to 1.80) between the overweight/obese participants and the normal weight ones. Both effects were large. For daily fruit intake, only normal weight participants showed a significant increase (1.15%, 95% CI: −2.85% to 5.16%, *p* = 0.022), with weight status effect (*p* = 0.803).

### 3.13 Correlation data

At baseline, body composition parameters including BMI, WC, %BF, and FM, were significantly and positively associated with BMR, LDL, TG, TC, HbA1c, fasting insulin, HOMA-IR, CRP. VO_2max_ was significantly associated with weight (r = −0.221), BMI (r = 0.209), FFM (r = 0.228), MVPA (r = 0.398) and HR_resting_ (r = −0.339). Body composition variables were highly correlated with each other. Regarding to PA data, MVPA was significantly associated with TPA (r = 0.406), HR_resting_ (r = −0.186), SBP (r = −0.295), HDL (r = 0.630), LDL (r = 0.210), and TC (r = 0.203). While TPA was only observed to be significantly associated with HDL (r = 0.391). For dietary data, daily energy intake was significantly associated with weight (r = 0.382), BMI (r = 0.458), WC (r = 0.511), %BF (r = 0.273), FM (r = 0.369), FFM (r = 328), BMR (r = 0.222), DBP (r = −0.199), LDL (r = 0.314), TG (r = 0.220), TC (r = 0.328), HbA1c (r = 0.252), fasting insulin (r = 0.209), HOMA-IR (r = 0.212) and CRP (r = 0.235). Vegetable intake was significantly associated with SBP (r = −0.337) and HDL (r = 0.188). There were significant and negative associations between fruit intake and CRP (r = −0.211). It should be noted that there was a strong correlation between the percentage change of MVPA and the percentage of TPA (r = 0.936, *p* < 0.001).

Correlation data was used to determine the highly collinear variables, which were removed from the regression model to avoid potential multicollinearity.

### 3.14 Regression analysis

According to the literature review, age, body composition (weight, BMI, %BF, FM, and FFM), cardiorespiratory fitness (VO_2max_), PA (MVPA and TPA), and dietary intake (daily energy intake, daily vegetable, and fruit intake) were all included in the regression model. Additionally, to evaluate the effect of the intervention, both the baseline value and the percentage change of covariables were included in the model. We also adjusted for the baseline value of cardiometabolic outcomes.

Regression analysis for cardiometabolic indicators were outlined in Table 4. In Model 1, after controlling for potential variables, neither the baseline value of MVPA nor TPA were associated with the percentage change of SBP (ΔSBP), HDL (ΔHDL), LDL (ΔLDL), TG (ΔTG), or TC (ΔTC). The baseline value of MVPA was significantly associated with the percentage change of fasting insulin (Δfasting insulin) (b = 0.278, *p* = 0.049) and HOMA-IR (ΔHOMA-IR) (b = 0.287, *p* = 0.020).

**TABLE 4:** Regression data.

Dependent variable		Independent variable	Standardized beta	*p*-value	Δ *R* ^2^
Δ HDL	Model 1: adjusted *R* ^2^ = 0.601	TPA	-0.171	0.112	
		MVPA	0.018	0.900	
	Model 2: adjusted *R* ^2^ = 0.692	TPA	0.121	0.323	
		MVPA	-0.040	0.751	
		Δ TPA	0.480	0.001	0.074
	Model 3: adjusted *R* ^2^ = 0.645	TPA	0.014	0.912	
		MVPA	-0.052	0.704	
		Δ MVPA	0.326	0.015	0.039
Model 1: adjusted for age, BMI, ΔBMI, VO2max, ΔVO2max, energy intake, Δ energy intake, vegetable, Δ vegetable, fruit, Δ fruit, HDL					
Δ LDL	Model 1: adjusted *R* ^2^ = 0.228	TPA	0.060	0.683	
		MVPA	0.296	0.080	
	Model 2: adjusted *R* ^2^ = 0.251	TPA	0.127	0.503	
		MVPA	0.336	0.048	
		Δ TPA	-0.309	0.131	0.031
	Model 3: adjusted *R* ^2^ = 0.219	TPA	-0.021	0.911	
		MVPA	0.307	0.073	
		Δ MVPA	-0.309	0.131	0.007
Model 1: adjusted for age, BMI, ΔBMI, VO2max, ΔVO2max, energy intake, Δ energy intake, vegetable, Δ vegetable, fruit, Δ fruit, LDL					
Δ TG	Model 1: adjusted *R* ^2^ = 0.254	TPA	0.094	0.542	
		MVPA	0.087	0.546	
	Model 2: adjusted *R* ^2^ = 0.241	TPA	0.021	0.912	
		MVPA	0.109	0.490	
		Δ TPA	-0.116	0.604	0.004
	Model 3: adjusted *R* ^2^ = 0.237	TPA	0.106	0.566	
		MVPA	0.092	0.556	
		Δ MVPA	0.033	0.869	0.000
Model 1: adjusted for age, BMI, ΔBMI, VO2max, ΔVO2max, energy intake, Δ energy intake, vegetable, Δ vegetable, fruit, Δ fruit, TG					
ΔTC	Model 1: adjusted *R* ^2^ = 0.499	TPA	0.208	0.083	
		MVPA	0.051	0.702	
	Model 2: adjusted *R* ^2^ = 0.488	TPA	0.178	0.255	
		MVPA	0.059	0.669	
		Δ TPA	-0.049	0.767	0.001
	Model 3: adjusted *R* ^2^ = 0.499	TPA	0.298	0.049	
		MVPA	0.036	0.79	
		Δ MVPA	0.154	0.312	0.009
Model 1: adjusted for age, BMI, ΔBMI, VO2max, ΔVO2max, energy intake, Δ energy intake, vegetable, Δ vegetable, fruit, Δ fruit, TC					
Δ SBP	Model 1: adjusted *R* ^2^ = 0.780	TPA	0.003	0.965	
		MVPA	0.033	0.697	
	Model 2: adjusted *R* ^2^ = 0.788	TPA	0.110	0.281	
		MVPA	-0.001	0.993	
		Δ TPA	0.174	0.111	0.010
	Model 3: adjusted *R* ^2^ = 0.779	TPA	0.054	0.581	
		MVPA	0.024	0.777	
		Δ MVPA	0.087	0.392	0.003
Model 1: adjusted for age, BMI, ΔBMI, VO2max, ΔVO2max, energy intake, Δ energy intake, vegetable, Δ vegetable, fruit, Δ fruit, SBP					
Δ fasting insulin	Model 1: adjusted *R* ^2^ = 0.391	TPA	-0.139	0.287	
		MVPA	0.278	0.049	
	Model 2: adjusted *R* ^2^ = 0.388	TPA	-0.239	0.171	
		MVPA	0.302	0.037	
		Δ TPA	-0.170	0.381	0.008
	Model 3: adjusted *R* ^2^ = 0.400	TPA	-0.263	0.110	
		MVPA	0.295	0.037	
		Δ MVPA	-0.213	0.212	0.017
Model 1: adjusted for age, BMI, ΔBMI, VO2max, ΔVO2max, energy intake, Δ energy intake, vegetable, Δ vegetable, fruit, Δ fruit, fasting insulin					
Δ HOMA-IR	Model 1: adjusted *R* ^2^ = 0.539	TPA	-0.105	0.358	
		MVPA	0.287	0.020	
	Model 2: adjusted *R* ^2^ = 0.543	TPA	-0.217	0.150	
		MVPA	0.316	0.013	
		Δ TPA	-0.197	0.251	0.011
	Model 3: adjusted *R* ^2^ = 0.544	TPA	-0.207	0.147	
		MVPA	0.302	0.015	
		Δ MVPA	-0.178	0.23	0.012
Model 1: adjusted for age, BMI, ΔBMI, VO2max, ΔVO2max, energy intake, Δ energy intake, vegetable, Δ vegetable, fruit, Δ fruit, HOMA-IR					

Note: BMI, body mass index; DBP, diastolic blood pressure; HDL, high-density lipoprotein; HOMA-IR, homeostasis model assessment of insulin resistance; LDL, low-density lipoprotein; MVPA, moderate-to-vigorous intensity physical activity; SBP, systolic blood pressure; TC, total-cholesterol; TG, triglyceride; TPA, total physical activity; VO2max, maximal oxygen uptake.

In Model 2, adding the percentage change of TPA (ΔTPA) significantly increased the explained variation in ΔHDL to 69.2% (Adjusted *R* ^2^ = 0.692). ΔTPA was independently associated with ΔHDL (b = 0.480, *p* = 0.001), accounting for 7.4% of the variation. For every 1% increase in TPA, HDL improved by 0.5%. The baseline value of MVPA was significantly associated with ΔLDL (b = 0.336, *p* = 0.048), Δfasting insulin (b = 0.302, *p* = 0.037), and ΔHOMA-IR (b = 0.316, *p* = 0.013) in Model 2. While ΔTPA was not associated with ΔSBP, ΔLDL, ΔTG, or ΔTC.

In Model 3, the percentage change of MVPA (ΔMVPA), significantly increased the explained variation in ΔHDL to 64.5% (Adjusted *R* ^2^ = 0.645). ΔMVPA was independently associated with ΔHDL (b = 0.326, *p* = 0.015), accounting for 3.9% of the variation. For every 1% increase in MVPA, HDL improved by 0.398%. The baseline value of TPA was significantly associated with ΔTC (b = 0.298, *p* = 0.049) in model 3. Furthermore, the baseline level of MVPA was significantly associated with Δfasting insulin (b = 0.326, *p* = 0.015) and ΔHOMA-IR (b = 0.302, *p* = 0.015).

## 4 Discussion

Even though there is accumulating evidence for the health benefits of HIIT in adults, the most consistent benefits had been seen in improving cardiorespiratory fitness. The benefits of HIIT on cardiometabolic health remain controversial. Particularly, there was limited data on the effects of HIIT on habitual PA. Since PA was found to be associated with cardiometabolic risk factors, it remains unknown whether PA played a mediating role on the effectiveness of HIIT. Furthermore, the effectiveness of a Tabata-style functional HIIT utilizing very short intervals and the exercise modality other than running or cycling on favorable changes in health has not been fully explored. The primary aim of the present study, therefore, was to examine the effects of a 12-week Tabata-style functional HIIT program on cardiometabolic risk factors and PA levels in female university students. The Tabata-style functional HIIT involved eight bouts of 20-s “all-out” functional exercises, intermitted by 10-s rest between each bout. In terms of exercise adherence, only 1 student dropped out due to the hypoglycemia during the first session. There was a satisfied adherence that 98.33% of participants attended all sessions. The fidelity of the intervention, which was evaluated by heart rate responses during exercises, was largely upheld since a high intensity was delivered to all of the participants (between-subject SD), consistently throughout the 12-week intervention (within-subject SD). The variation in both HR_mean_ and HR_peak_ across different exercise sessions were small (0.24% for HR_mean_ and 0.30% for HR_peak_), indicating that the exercise remained relatively consistent across the sessions. Furthermore, we did not find any difference on both HR measures between sessions. This might have been due to the familiarization session prior to the intervention.

After the 12-week intervention, compared to the control group, favorable intervention effects were observed in the Tabata group on cardiorespiratory fitness, most variables of body composition, some outcomes of cardiometabolic risk factors, and daily MVPA and TPA. These findings extended beneficial effects reported by previous studies and moreover, they were of particular importance from a perspective of health promotion in emerging adults, since the increasing prevalence of MetS and physical inactivity were observed in this population (Ford et al., 2004; Hirode and Wong, 2020; Tcymbal et al., 2020). Collectively, the Tabata-style functional HIIT provided a feasible and effective strategy to improving young women’s cardiometabolic health and habitual PA in the university setting.

### 4.1 Cardiorespiratory fitness effect

The improvement in cardiorespiratory fitness measured by VO_2max_ after HIIT were consistently reported by previous studies (Batacan et al., 2017; Jelleyman et al., 2015; Kessler et al., 2012). It was supported by our finding that VO_2max_ increased by 12.87% ± 7.16% in the Tabata group after intervention. Likewise, previous studies based on young adults showed similar improvements. De Revere et al. (2021) investigated the effect of a 3-week cycling-based HIIT protocol in non-obese and inactive women. Participants were required to complete 8–10 sets of 1-min workout followed by 75-s recovery. After a total of nine sessions, VO_2max_ increased about 10%. In the study by Menz et al. (2019), following a 4-week Tabata-style HIIT protocol with a total of 14 sessions, participants (5 females and 2males) improved their VO_2max_ by 11% ± 7% (Menz et al., 2019). However, compared with our protocol, the exercise volume was higher in De Revere et al. (2021); Menz et al. (2019)’s protocols, which was about 16 min per session. With a higher training volume, it was not surprising that participants were able to benefit from the HIIT program with short duration. Indeed, evidence from a previous systematic review showed that the improvement in VO_2max_ can be achieved following 2 weeks of HIIT with few exercise sessions (Kessler et al., 2012). Although the longer intervention duration appeared to contribute to additional increases in VO_2max_ (Milanović et al., 2015), it was not true in the present study. One of the potential explanations were the low training volume of each session. In our protocol, the total workout duration per session was just 4 min, which was only a quarter of Menz et al. (2019)’s and one-fifth of De Revere et al. (2021)’s protocol. Moreover; Rosenblat et al. (2022) suggested that although the improvement in VO_2max_ seemed similar between protocols with different interval types, those with shorter intervals (2s–60s) were more likely to increase skeletal capillary density and mitochondrial respiration. This might facilitate the ultimate improvements in whole body exercise capacity and endurance in untrained people (Jacobs et al., 2013). A greater improvement of 18% was reported by Foster et al. (2015) after an 8-week (24 sessions) cycling-based Tabata. The original exercise intensity for Tabata training of 170% VO_2max_ was used. Although an additional increase in VO_2max_ were obtained under the high intensity stimulation, it resulted in a negative affective response in the participants (Foster et al., 2015). It was the fact that the feasibility of the original intensity of 170% VO_2max_ was questioned in the real-world setting. The result of this study revealed that Tabata protocol utilizing a modified lower intensity (83.24% of HR_max_) was an alternative to effectively improve cardiorespiratory fitness in untrained individuals.

In contrast, in the work by Islam er al. (2020), participants completed a conventional 4-min Tabata training 4 times a week for 4 weeks. The exercise modality was whole body functional exercises including burpee push-ups, mountain climber push-ups, jumping jacks and squat and thrusts. After intervention, no significant improvement on VO_2max_ was observed (Islam et al., 2020). This might be due to the short duration of the intervention, as well as the better cardiorespiratory fitness of participants at baseline. Baseline cardiorespiratory fitness and initial training status were found to be associated with the training effects (Milanović et al., 2015; Støren et al., 2017).

Several studies examined the effects on cardiorespiratory fitness in overweight and obese individuals. After a 5-week HIIT intervention with a total of 20 sessions, VO_2max_ increased by 7.9% in obese young women (Kong et al., 2016). In Kong et al. (2016)’s protocol, each session lasted 20 min and comprised of 60 repeats of 8-s cycling followed by 12-s rest. The HR_mean_ over the intervention was 164 ± 8 bpm (81% of age predicted HRmax). In the study by Hu et al. (2021), a high volume of HIIT protocol was used. Each session involved 4 min of cycling at 90% of VO_2max_ followed by 3 min rest for a total of 60 min, with a frequency of 3 times a week. Following 36 training sessions, the obese participants had a significant increase of 20% in VO_2max_ (Hu et al., 2021). The study be Sun et al. (2019) reported a greater improvement of 25% in VO_2max_ in overweight young females following a 12-week HIIT protocol with a frequency of 3 times per week. During each session, participants were required to complete nine sets of 4-min cycling (90% of VO_2max_) followed by 3-min rest (Sun et al., 2019). Despite the low volume in the present study, in line with previous studies, findings from our sub-group analysis revealed that overweight and obese participants had a significant increase in VO_2max_ after the intervention (16.98% ± 6.01%). Furthermore, we found that the percentage increase in relative VO_2max_ differed statistically significantly between elevated- and normal-weight participants. However, absolute VO_2max_ showed no differences between groups. This might be due to the larger decrease on weight in the overweight/obese group. There was limited data on the direct comparison of the effects on cardiorespiratory fitness between normal weight adults and overweight or obese adults. Findings from a systematic review and meta-analysis showed that short-term HIIT (<12 weeks) had a large effect on improving VO_2max_ in normal weight adults, while a medium effect in overweight or obese adults. Meta-analysis was also available for the effect of long-term HIIT (≥12 weeks) in overweight or obese adults and the pooled result showed a large effect in overweight or obese adults (Batacan et al., 2017). It suggested that the duration of intervention was positively associated with the effectiveness, at least for overweight or obese populations. When studying the mechanism associated with the improvements on VO_2max_, central factors and peripheral factors should be considered. After HIIT, plasma volume, lest ventricular mass, maximal stroke volume, and maximal cardiac output were increased. In addition to central adaptations, capillary density, maximal citrate synthase activity and mitochondrial respiration were increased (Rosenblat et al., 2022). These physiological adaptations were responsible for the improvements on VO_2max_.

### 4.2 Body composition effect

Despite the increasing popularity of HIIT for weight and fat loss, its effectiveness remains controversial. Our results demonstrated that a 12-week Tabata-style functional HIIT was effective on reducing %BF and FM and increasing FFM. There were no changes in weight, BMI, or WC. The results from the current study indicated that HIIT was effective for fat loss but not for weight loss. It was in line with previous studies (Macpherson et al., 2011; Zhang et al., 2021). Macpherson et al. (2011) used a typical running-based HIIT that involved four to six sets of 30-s sprint followed by 4-min recovery. After 6 weeks’s intervention (3 times per week), %BF and FM decreased significantly and FFM increased significantly, but body mass was unchanged (Macpherson et al., 2011). The fat-reducing effect was also supported by Zhang et al. (2021)’s study, in which the reductions in whole-body and regional FM were reported after 12-week’s HIIT intervention in obese young women (Zhang et al., 2021). This was in line with a previous systematic review and meta-analysis that HIIT were able to significantly reduce total, abdominal and visceral fat mass (Maillard et al., 2018). However, the authors indicated that the reduction in abdominal fat mass could only be detected by computed tomography scan or magnetic resonance imaging. This might account for the absence of significant change in WC in the present study. Furthermore, only the HIIT protocol utilizing the exercise modality of running or cycling were included, and our results expanded the knowledge of the efficacy of HIIT in reducing fat. Non-etheless, there was controversy over whether HIIT was effective in lowering fat. Proponents argued that HIIT increased both aerobic and anaerobic capacity, reduced insulin resistance, and thus increased fat oxidation (Boutcher, 2011). On the contrary, the counterargument was that when exercises were performed at an intensity of 85% of VO_2max_ or greater, fat had little to do with energy, and metabolic energy comes almost exclusively from the breakdown of sugars in the body (Achten and Jeukendrup, 2004; Venables et al., 2005). From the perspective of energy balance, we believed that without controlling total energy expenditure and intake, it was hard to determine whether the fat-lowering effect of HIIT was caused by training itself or by dietary intake or habitual physical activity. Well-controlled studies are expected in the future.

However, the simultaneous loss of weight and body fat after HIIT training has been reported in several studies. Trapp et al. (2008) investigated the effects of a 15-week cycling-based HIIT on fat loss in young normal weight women. Participants performed 8-s sprinting followed by 12-s recovery for 60 repeats. After a total of 45 session (20 min each session), the body mass, fat mass, and %BF were significantly reduced (Trapp et al., 2008). In the study by Tjønna et al. (2008), participants completed a total of 48 sessions of HIIT (90% of HR_max_) with the frequency of three sessions a week. After 16 weeks’ intervention, body mass and fat were significantly reduced (Tjønna et al., 2008). Given the fact that there was no change in energy intake between pre- and post-tests, the absence of reduction on body mass might be due to the following reasons: 1) the low exercise volume resulted in low energy expenditure, which was not sufficient to induce energy deficit and further reducing weight; 2) participants were normal weight at baseline and most favorable effects on body mass were observed in participants with overweight or obese (Tjønna et al., 2008; Martins et al., 2016; D’Amuri et al., 2021). It was supported by findings from our subgroup analysis that body mass, BMI and WC significantly decreased in overweight/obese participants after intervention whereas these variables did not change in normal weight participants. Although the weight-lowering effect of the Tabata-style functional HIIT was not significant in the present study, it appeared to be a time-efficient way to prevent the abnormal weight gain among freshmen. Moreover, results from the present study demonstrated that overweight/obesity had an effect on weight change but did not affect the association between Tabata training and weight change. The greater weight-lowering effect observed in overweight/obese participants might be attributed to their larger increase in PA and decrease in energy intake compared to normal weight counterparts. This was further reinforced by acknowledging that the role of exercise training in the maintenance or improvement of weight was predominantly influenced by the cumulative effect of energy deficit during the daily life (LaForgia et al., 2006).

On the contrary, a systematic review and meta-analysis evaluated the effect of low-volume HIIT on body composition and reported that improvements on body composition outcomes such as FM, %BF or FFM were hardly observed following low-volume HIIT (Sultana et al., 2019). In the present study, the favorable effects observed on some body composition measures might be partially explained by the increased daily MVPA and TPA. According to the meta-analytical findings from our previous study, there was a moderate correlation between TPA and %BF (Lu et al., 2022b). The study also indicated that the improvement on adiposity could be seen when PA performed at moderate or higher intensity. On the other hand, the adaptations of fat in response to low-volume HIIT suggested a different underlying mechanism for fat reduction with MICT. The fat reduction after low-volume HIIT was not likely dependent on the amount of energy expended during exercise sessions. This might be attributed to the larger improvement on the metabolic rate and fat expenditure post intervention, because the magnitude and duration of excess post-exercise oxygen consumption was greater after HIIT (LaForgia et al., 2006) and lipolytic hormones, such as catecholamines and growth hormone, have been reported to increase with exercise intensity (McMurray et al., 1987). Moreover, HIIT was found to elicit a larger elevation of plasma catecholamines compared with steady-state exercise. This potentially facilitated fat reduction after HIIT (Zouhal et al., 2008).

### 4.3 Cardiometabolic indicators

#### 4.3.1 Blood pressure

Aerobic exercise was well documented to reduce resting BP and was recommended in the primary and secondary prevention of CVDs (Cornelissen and Smart, 2013; Johnson et al., 2014). While there was emerging evidence from intervention studies that HIIT was effective on improving resting SBP (Nybo et al., 2010; Holloway et al., 2018; Aghaei Bahmanbeglou et al., 2019; de Oliveira et al., 2020) or both SBP and DBP (Ciolac et al., 2010; Hu et al., 2022). Results from the present study showed that only SBP was significantly decreased after 12-week intervention. This agreed with several previous studies.

In the work by Aghaei Bahmanbeglou et al. (2019), participants with stage 1 hypertension completed either short interval HIIT (work rest ratio: 30-s/30-s at 80%–100% of VO_2max_) or long interval HIIT (work rest ratio: 4-min/4-min at 75%–90% of VO_2max_) for a total of 8 weeks. After the intervention, SBP was significantly decreased in both short interval HIIT and long interval HIIT, suggesting that the SBP-lowing effect of HIIT was irrespective of the intensity and exercise interval (Aghaei Bahmanbeglou et al., 2019). Similarly, de Oliveira et al. (2020) also reported a significant decrease in SBP but not in DBP after an 8-week’s HIIT intervention in young obese women with elevated BP at baseline. The training protocol involved four bouts of 4-min high-intensity running at 85%–95% of HRmax, followed by 3-min active recovery at 65–75 of HRmax (de Oliveira et al., 2020). It seemed that HIIT was effective in improving SBP in young and middle-aged individuals with abnormal SBP. It was supported by a recent systematic review and meta-analysis by Costa Lêdo et al. (2018). The authors reported that HIIT was equally effective in reducing BP compared to MICT in participants with pre- and established hypertension (Costa Lêdo et al., 2018). Nevertheless, inconsistent with our results, this systematic review suggested that DBP could also be improved by HIIT. The baseline value of DBP might explain the inconsistence because higher baseline values were more likely to be improved by exercises (Bravata et al., 2007).

In the subgroup analysis, we observed significant decreases in both overweight/obese group and normal weight group and there was no between-group difference. Most studies evaluated the effectiveness of HIIT in overweight or obese participants. A systematic review and meta-analysis examined the effect of HIIT in overweight/obese and normal weight population (Batacan et al., 2017). The results indicated the difference in the effect of HIIT on BP between participants with different BMI. The BP-lowing effect of HIIT was only observed in overweight/obese participants. However, our results showed that normal weight participants with elevated SBP could also benefit from HIIT. This was confirmed by previous studies that the degree of BP reduction was related to its baseline value (Pescatello et al., 2004; Bravata et al., 2007). A greater reduction on BP were found in participants with higher baseline BP readings.

However, a recent study provided opposite results that functional HIIT was not effective in reducing BP (Nunes et al., 2022). In Nunes et al. (2022)’s protocol, participants were required to complete 10 sets of 60-s of functional exercise followed by 60-s active recovery. After 12 weeks’ intervention (36 sessions), neither SBP nor DBP were improved significantly. The lack of a significant reduction in BP might be explained by the age of participants. In Nunes et al. (2022)’s study, participants were postmenopausal women with the mean age of 61.5 years, whereas participants in our study were young females with the age of 20.42 years. On one hand, SBP and DBP increased with age (Landahl et al., 1986). On the other hand, postmenopausal women were at high risk of hypertension due to the decline in estrogen (Saeed et al., 2017). Collectively, it seemed reasonable that post-intervention BP was not improved in older women after low-volume HIIT.

In general, BP improvements were more likely to be observed in aerobic, resistance and concurrent training (moderate-intensity aerobic exercise and high intensity resistance exercise) with a volume of 150 min per week (Sabbahi et al., 2016; Corso et al., 2016; Son et al., 2017). Our finding supported the favorable effect on SBP following low-volume HIIT. This favorable change might be due to the high intensity achieved during exercises (Eicher et al., 2010). Higher intensity was reported to be associated with greater acute reduction on BP following exercises, which contributed to chronic BP lowing responses (Liu et al., 2012). From a physiological perspective, several mechanisms had been proposed for BP reduction after aerobic training, such as improved vascular function, lowered inflammation, and oxidative stress. The work by Sawyer et al. (2016) suggested different vascular adaptations between HIIT and MICT (Sawyer et al., 2016). HIIT was found to increase brachial artery flow-mediated dilation (Tjønna et al., 2008) while MICT increased resting artery diameter and low flow-mediated constriction. These vascular adaptations could occur without improvements in body composition, which further supported our findings. Although only an improvement in SBP was detected after the intervention, it had important implications for CVD risk factors management, as a 10 mm Hg increase in SBP during young adulthood was found to be associated with a 14% increased risk of CVD mortality over a 41-year follow-up (McCarron et al., 2000). However, whether such HIIT protocol could be used in the clinical setting to improve the BP in participants with established hypertension need further investigations.

#### 4.3.2 Lipid profiles

Our findings suggested favorable effects on HDL, LDL, and TC after intervention, while all these improvements were observed in participants with overweight and obesity. We also found intervention × weight status interaction effects for LDL, TC and TG. This might be due to the higher pre-test value involved in the overweight/obese group. Our findings were in line with a previous study by Tjønna et al. (2008), in which HDL increased significantly after a 16-week (48 sessions) HIIT with an exercise intensity of 90% of HR_max_ in overweight/obese participants (Tjønna et al., 2008). Our findings were partly supported by a systematic review and meta-analysis that neither short-term nor long-term HIIT had significant effects on cardiometabolic risk factors in normal weight participants (Batacan et al., 2017). However, the authors indicated that the lipid profile was not improved in overweight/obese participants neither. Findings from another systematic review reported the same results that TC, TG, HDL, or LDL were not improvement after HIIT (Kessler et al., 2012).

Few studies examined effects on lipid biomarkers after HIIT in normal weight participants. Nevertheless, normal weight obesity had gained increasing attention in recent years and a study by Hu et al. (2022) investigated the effects of HIIT in this population. The HIIT protocol used in Hu et al. (2022)’s study involved three sets of 9-min workout at 90% of HR_max_ followed by 1-min rest, with a high frequency of 5 days per week. After 4 weeks’ training, TC, TG, LDL and HDL were significantly improved in young women with NOW (Hu et al., 2022). Although the intervention duration was only 4 weeks, which was one-third of that in the present study, its training volume was as high as 1350 MET-min/week. This volume was sufficient to see a meaningful amelioration in lipid levels (Mann et al., 2014).

Most beneficial effects of HIIT on lipid profiles were reported among overweight/obese men. In the work by Fisher et al. (2015), after 6 weeks’ intervention, TC, TG, LDL, and HDL were improved in young men with overweight or obesity (Fisher et al., 2015). A previous study reported similar results that HDL increased after an 8-week HIIT program in untrained young men. However, TC was unchanged. It was believed that HDL was the most easily improved lipid profile component from exercise (Mann et al., 2014). This was supported by evidence from the study by Nybo et al. (2010). The authors indicated that TC/HDL ratio was the only index that improved significantly after 150 min of MICT weekly at 65% of VO_2max_ for 12 weeks in untrained young men. Additionally, the authors compared the MICT with HIIT (40 min workout at 95% HR_max_ weekly) and there were no improvements in lipid profiles after HIIT (Nybo et al., 2010). This suggests that the volume of exercise, rather than the intensity of exercise, was the key to improving blood lipids and a relationship between body composition (body mass and %BF decreased only in MICT) and blood lipids was proposed. Similar findings were reported by Ho et al. (2012) that only combination exercise, after which weight, %BF, and FM decreased, had a beneficial effect on lipid profiles including TG, TC, HDL, and LDL (Ho et al., 2012). This was supported by a previous systematic review that weight loss provided significant favorable changes on blood lipid (Aucott et al., 2011). Therefore, despite the low training volume, the favorable effects on blood lipid observed in our study might be due to the reduction on body mass and fat.

#### 4.3.3 Carbohydrate metabolism and fasting insulin

Findings from the current study revealed that FPG and HbA1c were not significantly decreased after the Tabata-style functional HIIT, but HOMA-IR and fasting insulin decreased significantly. The improvements on fasting insulin and HOMA-IR observed in the present study were supported by a previous systematic review and meta-analysis (Jelleyman et al., 2015). Jelleyman et al. (2015) evaluated the effects of HIIT on biomarkers of glucose regulation and insulin resistance and meta-analytical findings demonstrated that compared to the control and MICT, insulin resistance significantly reduced following HIIT. The significant reduction on FPG only occurred in participants with elevated FPG value or diagnosed type 2 diabetes. This may help to explain the lack of advancement of FPG reduction in the present study as the baseline FPG value was normal in our samples. In contrast to our finding, HbA1c decreased significantly following HIIT compared to control. Another systematic review and meta-analysis provided similar finding related to insulin resistance, but FPG was not improved after HIIT (Kessler et al., 2012). These results appeared to be more consistent with the amelioration of insulin resistance following HIIT. Particularly, a previous study by Babraj et al. (2009) indicated that insulin sensitivity was improved following as short as 2 weeks of HIIT in normal weight adults. Unfortunately, this conclusion was based on males exclusively (Babraj et al., 2009). Contrast to the present study, Arad et al. (2015) reported that insulin sensitivity was not changed significantly after a 14-week HIIT in overweight/obese women (Arad et al., 2015). This discrepancy might be due to fact that neither weight nor fat were reduced in participants from Arad et al. (2015)’s study. It was evidenced by other studies that exercise training did not increase insulin sensitivity without weight and fat loss, whether that weight and fat loss is exercise-induced or diet-related (Ross et al., 2000; Gillen et al., 2013). However, a recent study indicated that both exercise training and weight loss (diet-induced) interventions improved insulin sensitivity. Body weight and fat mass were not significantly changed in participants in the exercise training group (Ryan et al., 2021). The authors also suggested that there were differential effects on signaling pathways in skeletal muscle between the exercise training and the diet-predominated weight loss intervention. It was supposed that exercise had an independent mechanism for improving insulin sensitivity and, in addition, might increase insulin sensitivity by losing weight. The latter might be explained from the perspective of energy expenditure, as evidence from previous studies suggests that total energy expenditure rather than exercise intensity is key to stimulating insulin sensitivity (Mayer-Davis et al., 1998). Previous studies studied physiological and molecular responses to a low-volume HIIT. Results from the Parolin et al. (1999)’s study showed that, during 3 × 30s all-out cycling, glycogen phosphorylase is predominantly activated during the first 15s of the first bout (Parolin et al., 1999). The study by Metcalfe et al. (2015) substantiated that the glycogen degradation with HIIT incorporating 20s all-out sprint was similar to that observed with those involving prolonged intervals (Metcalfe et al., 2015). The glycogen degradation was associated with the activation of AMPK (McBride and Hardie, 2009), which further contributed to the increase in peroxisome proliferator-activated receptor gamma coactivator 1-alpha (PGC-1α) and glucose transporter 4 (GLUT 4) gene expressions (Gibala et al., 2009). As such, it remained unclear whether the improvement in insulin sensitivity was induced by the intervention. On the one hand, the intervention effect observed in our study did not control for changes of body weight. On the other hand, we did not control the timing of the post-test, which could occur 1–7 days after the last session. Since a previous study showed no difference on insulin sensitivity between pre-training and 4 days after 12 weeks of training (Ryan et al., 2020), early post-intervention measurement might contribute to a higher level of insulin sensitivity. In line with our subgroup’s finding in relation to FPG; Tjønna et al. (2008) reported that after 16-week HIIT (48 sessions), overweight/obese participants had improved FPG, but insulin was not changed significantly (Tjønna et al., 2008). While it should be noted that the overweight/obese participants recruited by Tjønna et al. (2008) had elevated FPG, which was not the case for our participants. Another study based on overweight/obese participants with normal FPG reported that FPG decreased after 5-week HIIT (20 sessions) in young women (Kong et al., 2016). In was consistent with findings from a previous systematic review and meta-analysis that FPG improved significantly after HIIT in overweight/obese participants (Kessler et al., 2012). The change on FPG after training appeared to be independent of the pre-training value, but rather related to overweight/obesity. Results from our study suggested that low-volume HIIT was effective in improving FPG for overweight/obese young women with normal FPG value. This finding was important as MHO was an instable and transient phenotype and individuals with MHO were more likely to develop CVD events in the future (Eckel et al., 2016).

### 4.4 Inflammation

It is well known that exercise could mitigate the deleterious effects of aging, not only by improving adiposity and mitochondrial function, the key to oxidative stress and inflammation, but also by enhancing the antioxidant and anti-inflammatory capacities (Sallam and Laher, 2016). Some researchers claimed that exercise performed at high intensities increased inflammation and oxidative stress rather than reduced them (Davies et al., 1982; Bergholm et al., 1999; Goto et al., 2003), while others suggested that acute bouts of exercise induced oxidative stress and inflammatory responses (Moldoveanu et al., 2001; Farias-Junior et al., 2019). Our results revealed that inflammation, measured by pro-inflammatory indices of CRP, was not significantly changed following a 12-week Tabata-style functional HIIT. Likewise, in the work by Allen et al. (2017), participants completed a 9-week cycling-based HIIT with a frequency of 3 times per week. There were no significant changes on inflammatory biomarkers including TNF-α and CRP after intervention (Allen et al., 2017). These were not surprising because on one hand, intense exercise induced oxidative stress was normally recovered within 24 h according to our previous systematic review (Lu et al., 2021); on the other hand, 12 weeks was not sufficient long to exert the long-term anti-oxidative and anti-inflammatory effects of exercise training. Long-term HIIT (12 months), accompanied by high levels of habitual PA was recommended to establish a significant anti-inflammatory effect (Balducci et al., 2010). Additionally, Balducci et al. (2010) claimed that exercise induced anti-inflammatory effect was independent of weight loss. This was further confirmed by the present study that although weight and fat reduced significantly among overweight/obese participants, their CRP did not change following intervention.

### 4.5 Physical activity

There was limited data on the effect of HIIT on habitual PA. According to previous study, short-term Tabata-style functional HIIT was able to increase MVPA and TPA (Lu et al., 2022a). The present study examined the long-term effect on PA and similar results were observed. This suggested that long-term low-volume HIIT did not induce compensatory movement behaviors such as decreasing habitual PA among young women. It might be due to the low energy expenditure during the exercises. Compared to the control group, whose daily PA was significantly decreased over 12 weeks, the Tabata-style functional HIIT seemed to be a time-efficient way to promote habitual PA in the university setting. Furthermore, we also found a high correlation between the increase in MVPA and TPA, suggesting that the majority of TPA increased over 12 weeks was accumulated from the increase in MVPA. Although all participants in our study met the PA recommendation for health maintenance, MVPA that higher than 300 min weekly could provide additional health benefits (Bull et al., 2020). Surprisingly, we found intervention × weight status interactions for MVPA and TPA. There were greater increases on MVPA and TPA in overweight/obese participants compared to normal weight counterparts. It was hard to explain. This might be explained by several psychological changes. The intervention might have a greater effect on the autonomous motivation and exercise in overweight/obese women, facilitating the internalization of exercise behavioral regulation (Silva et al., 2011). Comparative studies were warranted in the future. Moreover, to our knowledge, this was the first study to examine the association between changes in PA and in cardiometabolic outcomes. Our hypothesis that there was a positive relationship between changes in cardiometabolic outcomes and changes in PA was partially confirmed. Results from the regression analysis showed that increasing TPA and MVPA were both independently associated with improvements on HDL. However, other cardiometabolic indicators showed no associations with improvements in MVPA or TPA. Surprisingly, we found a positive association between the baseline value of MVPA and the percentage of fasting insulin and HOMA-IR, indicating that participants with higher level of daily MVPA had greater increase in fasting insulin and HOMA-IR. It was hard to explain and might be due to the statistical error. Evidence from cross-sectional studies revealed that MVPA was negatively associated with fasting insulin and HOMA-IR values (Green et al., 2014). Therefore, the lower baseline value of fasting insulin and HOMA-IR had the potential to result larger percentage change, and the slight increase in post-test values were induced by the measurement errors. We also found a positive relationship between the baseline value of TPA and ΔTC. It might be related to the dietary pattern which was not examined in the present study.

## 5 Limitations

This was the first study to evaluate the effects of a Tabata-style functional HIIT on multiple cardiometabolic outcomes and physical activity in university female students. The strengths of our study included the randomized controlled design with a relatively large sample, supervised exercise training and robust measures of cardiometabolic biomarkers in a clinical setting. There were several Limitations that should be noted. Firstly, it was noted that there were associations between dietary intake and multiple investigated cardiometabolic measures, however, the nature of these associations was not fully investigated and well controlled in this study. Secondly, the present study was conducted based on a population who were more likely to gain weight during their first year of university. Such weight gain was not only associated with lifestyle changes, but also with several psychological factors such as perceived stress (Economos et al., 2008; Hootman et al., 2018) and the influence of peers (Smith-Jackson and Reel, 2012). Therefore, future studies aimed at weight management, psychological factors should be taken into consideration. Furthermore, the inter-individual variability for exercise fidelity was not further investigated and might have limited the ability to evaluate the intervention effects. Finally, although long-term HIIT (≥12 weeks) were recommended to evaluate the effects of HIIT, the duration of 12 weeks was still too short to evaluate clinical changes and sustainability in certain physiological and cardiometabolic health outcomes. Particularly, due to the lack of follow-up, we were unable to determine whether participants were willing or able to commit to such low-volume HIIT for a long period of time. Future research needs to be rigorously designed to include follow-up measures that will confidently assist policymakers in recommending the Tabata-style functional HIIT to promote health in the university setting.

## 6 Conclusion

The findings of the present study demonstrated that a 12-week Tabata-style functional exercises based HIIT intervention improved the cardiorespiratory fitness, body composition, some cardiometabolic biomarkers (SBP, HDL, LDL, TC, fasting insulin, and HOMA-IR), as well as daily habitual PA (MVPA and TPA) in female freshmen. Most health benefits in relation to body composition and cardiometabolic risk factors were observed in overweight/obese individuals. Furthermore, this study extends the current knowledge, by showing that increases in habitual PA following intervention were associated with a greater improvement on HDL post intervention.

## Data Availability

The original contributions presented in the study are included in the article/Supplementary Material, further inquiries can be directed to the corresponding author.

## References

[B1] Achten J. Jeukendrup A. E. (2004). Relation between plasma lactate concentration and fat oxidation rates over a wide range of exercise intensities. Int. J. Sports Med. 25 (1), 32–37. 10.1055/s-2003-45231 14750010

[B2] Aghaei Bahmanbeglou N. Ebrahim K. Maleki M. Nikpajouh A. Ahmadizad S. (2019). Short-duration high-intensity interval exercise training is more effective than long duration for blood pressure and arterial stiffness but not for inflammatory markers and lipid profiles in patients with stage 1 hypertension. J. Cardiopulm. Rehabil. Prev. 39 (1), 50–55. 10.1097/hcr.0000000000000377 30586113

[B3] Ajlouni K. Khader Y. Alyousfi M. Al Nsour M. Batieha A. Jaddou H. (2020). Metabolic syndrome amongst adults in Jordan: Prevalence, trend, and its association with socio-demographic characteristics. Diabetology metabolic syndrome 12 (1), 100. 10.1186/s13098-020-00610-7 33292456 PMC7672879

[B4] Alberti K. G. Eckel R. H. Grundy S. M. Zimmet P. Z. Cleeman J. I. Donato K. A. (2009). Harmonizing the metabolic syndrome: A joint interim statement of the international diabetes federation task force on epidemiology and prevention; national heart, lung, and blood institute; American heart association; world heart federation; international atherosclerosis society; and international association for the study of obesity. Circulation 120 (16), 1640–1645. 10.1161/circulationaha.109.192644 19805654

[B5] Allen N. G. Higham S. M. Mendham A. E. Kastelein T. E. Larsen P. S. Duffield R. (2017). The effect of high-intensity aerobic interval training on markers of systemic inflammation in sedentary populations. Eur. J. Appl. Physiol. 117 (6), 1249–1256. 10.1007/s00421-017-3613-1 28409397

[B6] Andersen L. B. Hasselstrøm H. Grønfeldt V. Hansen S. E. Karsten F. (2004). The relationship between physical fitness and clustered risk, and tracking of clustered risk from adolescence to young adulthood: Eight years follow-up in the Danish youth and sport study. Int. J. Behav. Nutr. Phys. Act. 1 (1), 6. 10.1186/1479-5868-1-6 15169561 PMC416568

[B7] Arad A. D. DiMenna F. J. Thomas N. Tamis-Holland J. Weil R. Geliebter A. (2015). High-intensity interval training without weight loss improves exercise but not basal or insulin-induced metabolism in overweight/obese African American women. J. Appl. Physiol. 119 (4), 352–362. 10.1152/japplphysiol.00306.2015 26112241

[B8] Arnold N. Lechner K. Waldeyer C. Shapiro M. D. Koenig W. (2021). Inflammation and cardiovascular disease: The future. Eur. Cardiol. 16, e20. 10.15420/ecr.2020.50 34093741 PMC8157394

[B9] Aucott L. Gray D. Rothnie H. Thapa M. Waweru C. (2011). Effects of lifestyle interventions and long-term weight loss on lipid outcomes - a systematic review. Obes. Rev. 12 (5), e412–e425. 10.1111/j.1467-789X.2010.00819.x 21371252

[B10] Babraj J. A. Vollaard N. B. Keast C. Guppy F. M. Cottrell G. Timmons J. A. (2009). Extremely short duration high intensity interval training substantially improves insulin action in young healthy males. BMC Endocr. Disord. 9, 3. 10.1186/1472-6823-9-3 19175906 PMC2640399

[B11] Balducci S. Zanuso S. Nicolucci A. Fernando F. Cavallo S. Cardelli P. (2010). Anti-inflammatory effect of exercise training in subjects with type 2 diabetes and the metabolic syndrome is dependent on exercise modalities and independent of weight loss. Nutr. Metab. Cardiovasc Dis. 20 (8), 608–617. 10.1016/j.numecd.2009.04.015 19695853

[B12] Batacan R. B. Jr. Duncan M. J. Dalbo V. J. Tucker P. S. Fenning A. S. (2017). Effects of high-intensity interval training on cardiometabolic health: A systematic review and meta-analysis of intervention studies. Br. J. Sports Med. 51 (6), 494–503. 10.1136/bjsports-2015-095841 27797726

[B13] Bergholm R. Mäkimattila S. Valkonen M. Liu M. L. Lahdenperä S. Taskinen M. R. (1999). Intense physical training decreases circulating antioxidants and endothelium-dependent vasodilatation *in vivo* . Atherosclerosis 145 (2), 341–349. 10.1016/s0021-9150(99)00089-1 10488962

[B14] Boutcher S. H. (2011). High-intensity intermittent exercise and fat loss. J. Obes. 2011, 868305. 10.1155/2011/868305 21113312 PMC2991639

[B15] Bravata D. M. Smith-Spangler C. Sundaram V. Gienger A. L. Lin N. Lewis R. (2007). Using pedometers to increase physical activity and improve health: A systematic review. Jama 298 (19), 2296–2304. 10.1001/jama.298.19.2296 18029834

[B16] Brown D. M. Bray S. R. Beatty K. R. Kwan M. Y. (2014). Healthy active living: A residence community-based intervention to increase physical activity and healthy eating during the transition to first-year University. J. Am. Coll. Health 62 (4), 234–242. 10.1080/07448481.2014.887572 24499161

[B17] Bull F. C. Al-Ansari S. S. Biddle S. Borodulin K. Buman M. P. Cardon G. (2020). World Health Organization 2020 guidelines on physical activity and sedentary behaviour. Br. J. Sports Med. 54 (24), 1451–1462. 10.1136/bjsports-2020-102955 33239350 PMC7719906

[B18] Casas M. Chatzi L. Carsin A. E. Amiano P. Guxens M. Kogevinas M. (2013). Maternal pre-pregnancy overweight and obesity, and child neuropsychological development: Two southern European birth cohort studies. Int. J. Epidemiol. 42 (2), 506–517. 10.1093/ije/dyt002 23569191

[B19] Castillo I. Solano S. Sepúlveda A. R. (2019). A controlled study of an integrated prevention program for improving disordered eating and body image among Mexican University students: A 3-month follow-up. Eur. Eat. Disord. Rev. 27 (5), 541–556. 10.1002/erv.2674 30997721

[B20] Chiang T. L. Chen C. Hsu C. H. Lin Y. C. Wu H. J. (2019). Is the goal of 12,000 steps per day sufficient for improving body composition and metabolic syndrome? The necessity of combining exercise intensity: A randomized controlled trial. BMC Public Health 19 (1), 1215. 10.1186/s12889-019-7554-y 31481039 PMC6724241

[B21] Ciolac E. G. Bocchi E. A. Bortolotto L. A. Carvalho V. O. Greve J. M. Guimarães G. V. (2010). Effects of high-intensity aerobic interval training vs. moderate exercise on hemodynamic, metabolic and neuro-humoral abnormalities of young normotensive women at high familial risk for hypertension. Hypertens. Res. 33 (8), 836–843. 10.1038/hr.2010.72 20448634

[B22] Cnattingius S. Villamor E. Johansson S. Edstedt Bonamy A. K. Persson M. Wikström A. K. (2013). Maternal obesity and risk of preterm delivery. Jama 309 (22), 2362–2370. 10.1001/jama.2013.6295 23757084

[B23] Cornelissen V. A. Smart N. A. (2013). Exercise training for blood pressure: A systematic review and meta-analysis. J. Am. Heart Assoc. 2 (1), e004473. 10.1161/jaha.112.004473 23525435 PMC3603230

[B24] Corso L. M. Macdonald H. V. Johnson B. T. Farinatti P. Livingston J. Zaleski A. L. (2016). Is concurrent training efficacious antihypertensive therapy? A meta-analysis. Med. Sci. Sports Exerc 48 (12), 2398–2406. 10.1249/mss.0000000000001056 27471784

[B25] Costa Lêdo V. R. Xavier A. P. de Souza C. A. Z. Fernandes S. M. S. Rodrigues É. Caperuto É C. (2018). Aquatic myofascial release applied after high intensity exercise increases flexibility and decreases pain. J. Bodyw. Mov. Ther. 22 (1), 97–104. 10.1016/j.jbmt.2017.05.013 29332765

[B26] D'Amuri A. Sanz J. M. Capatti E. Di Vece F. Vaccari F. Lazzer S. (2021). Effectiveness of high-intensity interval training for weight loss in adults with obesity: A randomised controlled non-inferiority trial. BMJ Open Sport Exerc Med. 7 (3), e001021. 10.1136/bmjsem-2020-001021 PMC8292807 34367654

[B27] Davies K. J. Quintanilha A. T. Brooks G. A. Packer L. (1982). Free radicals and tissue damage produced by exercise. Biochem. Biophys. Res. Commun. 107 (4), 1198–1205. 10.1016/s0006-291x(82)80124-1 6291524

[B28] de Oliveira G. H. Boutouyrie P. Simões C. F. Locatelli J. C. Mendes V. H. S. Reck H. B. (2020). The impact of high-intensity interval training (HIIT) and moderate-intensity continuous training (MICT) on arterial stiffness and blood pressure in young obese women: A randomized controlled trial. Hypertens. Res. 43 (11), 1315–1318. 10.1038/s41440-020-0477-2 32467641

[B29] De Revere J. L. Clausen R. D. Astorino T. A. (2021). Changes in VO2max and cardiac output in response to short-term high-intensity interval training in caucasian and hispanic young women: A pilot study. PLoS One 16 (1), e0244850. 10.1371/journal.pone.0244850 33481836 PMC7822506

[B30] Dickie K. Micklesfield L. K. Chantler S. Lambert E. V. Goedecke J. H. (2014). Meeting physical activity guidelines is associated with reduced risk for cardiovascular disease in black South African women; a 5.5-year follow-up study. BMC Public Health 14, 498. 10.1186/1471-2458-14-498 24886324 PMC4051116

[B31] Dishman R. K. (1994). The measurement conundrum in exercise adherence research. Med. Sci. Sports Exerc 26 (11), 1382–1390. 10.1249/00005768-199411000-00013 7837959

[B32] Domaradzki J. Cichy I. Rokita A. Popowczak M. (2020). Effects of Tabata training during physical education classes on body composition, aerobic capacity, and anaerobic performance of under-normal- and overweight adolescents. Int. J. Environ. Res. Public Health 17 (3), 876. 10.3390/ijerph17030876 32019253 PMC7038039

[B33] Eather N. Riley N. Miller A. Smith V. Poole A. Vincze L. (2019). Efficacy and feasibility of HIIT training for University students: The Uni-HIIT RCT. J. Sci. Med. Sport 22 (5), 596–601. 10.1016/j.jsams.2018.11.016 30509862

[B34] Eckel N. Meidtner K. Kalle-Uhlmann T. Stefan N. Schulze M. B. (2016). Metabolically healthy obesity and cardiovascular events: A systematic review and meta-analysis. Eur. J. Prev. Cardiol. 23 (9), 956–966. 10.1177/2047487315623884 26701871

[B160] Economos C.D. Hildebrandt M.L. Hyatt R.R. (2008). College freshman stress and weight change: differences by gender. Am J Health Behav . 32 (1), 16–25. 10.5555/ajhb.2008.32.1.16 18021030

[B35] Eicher J. D. Maresh C. M. Tsongalis G. J. Thompson P. D. Pescatello L. S. (2010). The additive blood pressure lowering effects of exercise intensity on post-exercise hypotension. Am. Heart J. 160 (3), 513–520. 10.1016/j.ahj.2010.06.005 20826261

[B36] El Ansari W. Stock C. Mikolajczyk R. T. (2012). Relationships between food consumption and living arrangements among University students in four European countries - a cross-sectional study. Nutr. J. 11, 28. 10.1186/1475-2891-11-28 22531503 PMC3420253

[B37] Farias-Junior L. F. Browne R. A. V. Freire Y. A. Oliveira-Dantas F. F. Lemos T. Galvão-Coelho N. L. (2019). Psychological responses, muscle damage, inflammation, and delayed onset muscle soreness to high-intensity interval and moderate-intensity continuous exercise in overweight men. Physiol. Behav. 199, 200–209. 10.1016/j.physbeh.2018.11.028 30471384

[B38] Fisher G. Brown A. W. Bohan Brown M. M. Alcorn A. Noles C. Winwood L. (2015). High intensity interval-vs moderate intensity- training for improving cardiometabolic health in overweight or obese males: A randomized controlled trial. PLoS One 10 (10), e0138853. 10.1371/journal.pone.0138853 26489022 PMC4619258

[B39] Fletcher G. F. Ades P. A. Kligfield P. Arena R. Balady G. J. Bittner V. A. (2013). Exercise standards for testing and training: A scientific statement from the American heart association. Circulation 128 (8), 873–934. 10.1161/CIR.0b013e31829b5b44 23877260

[B40] Follador L. Alves R. C. Ferreira S. D. S. Buzzachera C. F. Andrade V. Garcia E. (2018). Physiological, perceptual, and affective responses to six high-intensity interval training protocols. Percept. Mot. Ski. 125 (2), 329–350. 10.1177/0031512518754584 29368530

[B41] Ford E. S. Giles W. H. Mokdad A. H. (2004). Increasing prevalence of the metabolic syndrome among u.s. Adults. Diabetes Care 27 (10), 2444–2449. 10.2337/diacare.27.10.2444 15451914

[B42] Foster C. Farland C. V. Guidotti F. Harbin M. Roberts B. Schuette J. (2015). The effects of high intensity interval training vs steady state training on aerobic and anaerobic capacity. J. Sports Sci. Med. 14 (4), 747–755. 26664271 PMC4657417

[B43] Garber C. E. Blissmer B. Deschenes M. R. Franklin B. A. Lamonte M. J. Lee I. M. (2011). American college of sports medicine position stand. Quantity and quality of exercise for developing and maintaining cardiorespiratory, musculoskeletal, and neuromotor fitness in apparently healthy adults: Guidance for prescribing exercise. Med. Sci. Sports Exerc 43 (7), 1334–1359. 10.1249/MSS.0b013e318213fefb 21694556

[B44] Gellish R. L. Goslin B. R. Olson R. E. McDonald A. Russi G. D. Moudgil V. K. (2007). Longitudinal modeling of the relationship between age and maximal heart rate. Med. Sci. Sports Exerc 39 (5), 822–829. 10.1097/mss.0b013e31803349c6 17468581

[B45] Gentil P. Naves J. P. Viana R. B. Coswig V. Dos Santos Vaz M. Bartel C. (2016). Revisiting tabata's protocol: Does it even exist? Med. Sci. Sports Exerc 48 (10), 2070–2071. 10.1249/mss.0000000000001023 27635774

[B46] Gibala M. J. McGee S. L. Garnham A. P. Howlett K. F. Snow R. J. Hargreaves M. (2009). Brief intense interval exercise activates AMPK and p38 MAPK signaling and increases the expression of PGC-1alpha in human skeletal muscle. J. Appl. Physiol. 106 (3), 929–934. 10.1152/japplphysiol.90880.2008 19112161

[B47] Gillen J. B. Percival M. E. Ludzki A. Tarnopolsky M. A. Gibala M. J. (2013). Interval training in the fed or fasted state improves body composition and muscle oxidative capacity in overweight women. Obes. (Silver Spring) 21 (11), 2249–2255. 10.1002/oby.20379 23723099

[B48] Goto C. Higashi Y. Kimura M. Noma K. Hara K. Nakagawa K. (2003). Effect of different intensities of exercise on endothelium-dependent vasodilation in humans: Role of endothelium-dependent nitric oxide and oxidative stress. Circulation 108 (5), 530–535. 10.1161/01.Cir.0000080893.55729.28 12874192

[B49] Grasdalsmoen M. Eriksen H. R. Lønning K. J. Sivertsen B. (2019). Physical exercise and body-mass index in young adults: A national survey of Norwegian University students. BMC Public Health 19 (1), 1354. 10.1186/s12889-019-7650-z 31646998 PMC6813074

[B50] Green A. N. McGrath R. Martinez V. Taylor K. Paul D. R. Vella C. A. (2014). Associations of objectively measured sedentary behavior, light activity, and markers of cardiometabolic health in young women. Eur. J. Appl. Physiol. 114 (5), 907–919. 10.1007/s00421-014-2822-0 24463602

[B51] Gu Y. He Y. Ali S. H. Harper K. Dong H. Gittelsohn J. (2021). Fruit and vegetable intake and all-cause mortality in a Chinese population: The China health and nutrition survey. Int. J. Environ. Res. Public Health 18 (1), 342. 10.3390/ijerph18010342 33466375 PMC7794965

[B52] Haase A. Steptoe A. Sallis J. F. Wardle J. (2004). Leisure-time physical activity in University students from 23 countries: Associations with health beliefs, risk awareness, and national economic development. Prev. Med. 39 (1), 182–190. 10.1016/j.ypmed.2004.01.028 15208001

[B53] Heeren G. A. Jemmott J. B. 3rd Marange C. S. Rumosa Gwaze A. Batidzirai J. M. Ngwane Z. (2018). Health-promotion intervention increases self-reported physical activity in sub-saharan african university students: A randomized controlled pilot study. Behav. Med. 44 (4), 297–305. 10.1080/08964289.2017.1350134 28682186 PMC6292207

[B54] Hernández-Jaña S. Huber-Pérez T. Palma-Leal X. Guerrero-Ibacache P. Campos-Nuñez V. Zavala-Crichton J. P. (2020). Effect of a single nutritional intervention previous to a critical period of fat gain in university students with overweight and obesity: A randomized controlled trial. Int. J. Environ. Res. Public Health 17 (14), 5149. 10.3390/ijerph17145149 32708831 PMC7400622

[B55] Hirode G. Wong R. J. (2020). Trends in the prevalence of metabolic syndrome in the United States, 2011-2016. Jama 323 (24), 2526–2528. 10.1001/jama.2020.4501 32573660 PMC7312413

[B56] Ho S. S. Dhaliwal S. S. Hills A. P. Pal S. (2012). The effect of 12 weeks of aerobic, resistance or combination exercise training on cardiovascular risk factors in the overweight and obese in a randomized trial. BMC Public Health 12, 704. 10.1186/1471-2458-12-704 23006411 PMC3487794

[B57] Holloway K. Roche D. Angell P. (2018). Evaluating the progressive cardiovascular health benefits of short-term high-intensity interval training. Eur. J. Appl. Physiol. 118 (10), 2259–2268. 10.1007/s00421-018-3952-6 30078106

[B161] Hootman K.C. Guertin K.A. Cassano P.A. (2018). Stress and psychological constructs related to eating behavior are associated with anthropometry and body composition in young adults. Appetite. 125 287–294. 10.1016/j.appet.2018.01.003 29309851 PMC5878735

[B58] Hopkins W. G. Marshall S. W. Batterham A. M. Hanin J. (2009). Progressive statistics for studies in sports medicine and exercise science. Med. Sci. Sports Exerc 41 (1), 3–13. 10.1249/MSS.0b013e31818cb278 19092709

[B59] Hovell M. F. Mewborn C. R. Randle Y. Fowler-Johnson S. (1985). Risk of excess weight gain in University women: A three-year community controlled analysis. Addict. Behav. 10 (1), 15–28. 10.1016/0306-4603(85)90049-8 4003134

[B60] Hu J. Liu M. Yang R. Wang L. Liang L. Yang Y. (2022). Effects of high-intensity interval training on improving arterial stiffness in Chinese female University students with normal weight obese: A pilot randomized controlled trial. J. Transl. Med. 20 (1), 60. 10.1186/s12967-022-03250-9 35109880 PMC8809004

[B61] Hu M. Kong Z. Sun S. Zou L. Shi Q. Chow B. C. (2021). Interval training causes the same exercise enjoyment as moderate-intensity training to improve cardiorespiratory fitness and body composition in young Chinese women with elevated BMI. J. Sports Sci. 39 (15), 1677–1686. 10.1080/02640414.2021.1892946 33634738

[B62] Islam H. Siemens T. L. Matusiak J. B. L. Sawula L. Bonafiglia J. T. Preobrazenski N. (2020). Cardiorespiratory fitness and muscular endurance responses immediately and 2 months after a whole-body Tabata or vigorous-intensity continuous training intervention. Appl. physiology, Nutr. metabolism 45 (6), 650–658. 10.1139/apnm-2019-0492 31782930

[B63] Jacobs R. A. Flück D. Bonne T. C. Bürgi S. Christensen P. M. Toigo M. (2013). Improvements in exercise performance with high-intensity interval training coincide with an increase in skeletal muscle mitochondrial content and function. J. Appl. Physiol. 115 (6), 785–793. 10.1152/japplphysiol.00445.2013 23788574

[B64] Jang I. Kim J. S. (2019). Risk of cardiovascular disease related to metabolic syndrome in college students: A cross-sectional secondary data analysis. Int. J. Environ. Res. Public Health 16 (19), 3708. 10.3390/ijerph16193708 31581588 PMC6801641

[B65] Jelleyman C. Yates T. O'Donovan G. Gray L. J. King J. A. Khunti K. (2015). The effects of high-intensity interval training on glucose regulation and insulin resistance: A meta-analysis. Obes. Rev. 16 (11), 942–961. 10.1111/obr.12317 26481101

[B66] Johnson B. T. MacDonald H. V. Bruneau M. L. Jr. Goldsby T. U. Brown J. C. Huedo-Medina T. B. (2014). Methodological quality of meta-analyses on the blood pressure response to exercise: A review. J. Hypertens. 32 (4), 706–723. 10.1097/hjh.0000000000000097 24463936

[B67] Joshi D. Dodge T. (2022). Compensatory physical activity: Impact on type of physical activity and physical activity habits among female young adults. J. Am. Coll. Health 70 (1), 39–48. 10.1080/07448481.2020.1719113 32045337

[B68] Kaminsky L. A. Imboden M. T. Arena R. Myers J. (2017). Reference standards for cardiorespiratory fitness measured with cardiopulmonary exercise testing using cycle ergometry: Data from the fitness registry and the importance of exercise national database (FRIEND) registry. Mayo Clin. Proc. 92 (2), 228–233. 10.1016/j.mayocp.2016.10.003 27938891

[B69] Kass A. E. Jones M. Kolko R. P. Altman M. Fitzsimmons-Craft E. E. Eichen D. M. (2017). Universal prevention efforts should address eating disorder pathology across the weight spectrum: Implications for screening and intervention on college campuses. Eat. Behav. 25, 74–80. 10.1016/j.eatbeh.2016.03.019 27090854 PMC5042805

[B70] Kessler H. S. Sisson S. B. Short K. R. (2012). The potential for high-intensity interval training to reduce cardiometabolic disease risk. Sports Med. 42 (6), 489–509. 10.2165/11630910-000000000-00000 22587821

[B71] Kim S. Kim J. Y. Lee D. C. Lee H. S. Lee J. W. Jeon J. Y. (2014). Combined impact of cardiorespiratory fitness and visceral adiposity on metabolic syndrome in overweight and obese adults in Korea. PLoS One 9 (1), e85742. 10.1371/journal.pone.0085742 24454926 PMC3893257

[B72] King N. A. Hopkins M. Caudwell P. Stubbs R. J. Blundell J. E. (2008). Individual variability following 12 weeks of supervised exercise: Identification and characterization of compensation for exercise-induced weight loss. Int. J. Obes. (Lond) 32 (1), 177–184. 10.1038/sj.ijo.0803712 17848941

[B73] Kljajević V. Stanković M. Đorđević D. Trkulja-Petković D. Jovanović R. Plazibat K. (2021). Physical activity and physical fitness among university students-A systematic review. Int. J. Environ. Res. Public Health 19 (1), 158. 10.3390/ijerph19010158 35010418 PMC8750240

[B74] Kong Z. Sun S. Liu M. Shi Q. (2016). Short-term high-intensity interval training on body composition and blood glucose in overweight and obese young women. J. Diabetes Res. 2016, 4073618. 10.1155/2016/4073618 27774458 PMC5059579

[B75] Kushner R. F. Gudivaka R. Schoeller D. A. (1996). Clinical characteristics influencing bioelectrical impedance analysis measurements. Am. J. Clin. Nutr. 64 (3), 423s–427s. 10.1093/ajcn/64.3.423S 8780358

[B76] Kwan M. Y. Cairney J. Faulkner G. E. Pullenayegum E. E. (2012). Physical activity and other health-risk behaviors during the transition into early adulthood: A longitudinal cohort study. Am. J. Prev. Med. 42 (1), 14–20. 10.1016/j.amepre.2011.08.026 22176841

[B77] LaForgia J. Withers R. T. Gore C. J. (2006). Effects of exercise intensity and duration on the excess post-exercise oxygen consumption. J. Sports Sci. 24 (12), 1247–1264. 10.1080/02640410600552064 17101527

[B78] Lamoureux N. R. Fitzgerald J. S. Norton K. I. Sabato T. Tremblay M. S. Tomkinson G. R. (2019). Temporal trends in the cardiorespiratory fitness of 2,525,827 adults between 1967 and 2016: A systematic review. Sports Med. 49 (1), 41–55. 10.1007/s40279-018-1017-y 30390202

[B79] Lan C. Liu Y. Wang Y. (2022). Effects of different exercise programs on cardiorespiratory fitness and body composition in college students. J. Exerc Sci. Fit. 20 (1), 62–69. 10.1016/j.jesf.2021.12.004 35024049 PMC8724869

[B80] Landahl S. Bengtsson C. Sigurdsson J. A. Svanborg A. Svärdsudd K. (1986). Age-related changes in blood pressure. Hypertension 8 (11), 1044–1049. 10.1161/01.hyp.8.11.1044 3770866

[B81] Laursen P. B. Jenkins D. G. (2002). The scientific basis for high-intensity interval training: Optimising training programmes and maximising performance in highly trained endurance athletes. Sports Med. 32 (1), 53–73. 10.2165/00007256-200232010-00003 11772161

[B82] Lhakhang P. Godinho C. Knoll N. Schwarzer R. (2014). A brief intervention increases fruit and vegetable intake. A comparison of two intervention sequences. Appetite 82, 103–110. 10.1016/j.appet.2014.07.014 25049137

[B83] Li R. Li W. Lun Z. Zhang H. Sun Z. Kanu J. S. (2016). Prevalence of metabolic syndrome in mainland China: A meta-analysis of published studies. BMC Public Health 16, 296. 10.1186/s12889-016-2870-y 27039079 PMC4818385

[B84] Lim J. Park S. Kim J. S. (2021). Joint association of aerobic physical activity and muscle-strengthening activities with metabolic syndrome: The Korean national health and nutrition examination survey 2014-2015. Epidemiol. Health 43, e2021096. 10.4178/epih.e2021096 34773937 PMC8920739

[B85] Liu S. Goodman J. Nolan R. Lacombe S. Thomas S. G. (2012). Blood pressure responses to acute and chronic exercise are related in prehypertension. Med. Sci. Sports Exerc 44 (9), 1644–1652. 10.1249/MSS.0b013e31825408fb 22899388

[B86] Lovell G. P. El Ansari W. Parker J. K. (2010). Perceived exercise benefits and barriers of non-exercising female University students in the United Kingdom. Int. J. Environ. Res. Public Health 7 (3), 784–798. 10.3390/ijerph7030784 20617003 PMC2872307

[B87] Lu Y. Wiltshire H. D. Baker J. S. Wang Q. (2021). Effects of high intensity exercise on oxidative stress and antioxidant status in untrained humans: A systematic review. Biol. (Basel) 10 (12), 1272. 10.3390/biology10121272 PMC8698973 34943187

[B88] Lu Y. Wiltshire H. D. Baker J. S. Wang Q. (2022a). Effects of low-volume high-intensity interval exercise on 24 h movement behaviors in inactive female university students. Int. J. Environ. Res. Public Health 19 (12), 7177. 10.3390/ijerph19127177 35742425 PMC9223473

[B89] Lu Y. Wiltshire H. D. Baker J. S. Wang Q. Ying S. Li J. (2022b). Objectively determined physical activity and adiposity measures in adult women: A systematic review and meta-analysis. Front. Physiol. 13, 935892. 10.3389/fphys.2022.935892 36082217 PMC9445154

[B90] Lv N. Azar K. M. J. Rosas L. G. Wulfovich S. Xiao L. Ma J. (2017). Behavioral lifestyle interventions for moderate and severe obesity: A systematic review. Prev. Med. 100, 180–193. 10.1016/j.ypmed.2017.04.022 28450123 PMC5503454

[B91] Macpherson R. E. Hazell T. J. Olver T. D. Paterson D. H. Lemon P. W. (2011). Run sprint interval training improves aerobic performance but not maximal cardiac output. Med. Sci. Sports Exerc 43 (1), 115–122. 10.1249/MSS.0b013e3181e5eacd 20473222

[B92] Maillard F. Pereira B. Boisseau N. (2018). Effect of high-intensity interval training on total, abdominal and visceral fat mass: A meta-analysis. Sports Med. 48 (2), 269–288. 10.1007/s40279-017-0807-y 29127602

[B93] Mann S. Beedie C. Jimenez A. (2014). Differential effects of aerobic exercise, resistance training and combined exercise modalities on cholesterol and the lipid profile: Review, synthesis and recommendations. Sports Med. 44 (2), 211–221. 10.1007/s40279-013-0110-5 24174305 PMC3906547

[B94] Martins C. Kazakova I. Ludviksen M. Mehus I. Wisloff U. Kulseng B. (2016). High-intensity interval training and isocaloric moderate-intensity continuous training result in similar improvements in body composition and fitness in obese individuals. Int. J. Sport Nutr. Exerc Metab. 26 (3), 197–204. 10.1123/ijsnem.2015-0078 26479856

[B95] Maselli M. Ward P. B. Gobbi E. Carraro A. (2018). Promoting physical activity among university students: A systematic review of controlled trials. Am. J. Health Promot 32 (7), 1602–1612. 10.1177/0890117117753798 29366334

[B96] Mayer-Davis E. J. D'Agostino R. Jr. Karter A. J. Haffner S. M. Rewers M. J. Saad M. (1998). Intensity and amount of physical activity in relation to insulin sensitivity: The insulin resistance atherosclerosis study. Jama 279 (9), 669–674. 10.1001/jama.279.9.669 9496984

[B97] McBride A. Hardie D. G. (2009). AMP-activated protein kinase-a sensor of glycogen as well as AMP and ATP? Acta Physiol. (Oxf) 196 (1), 99–113. 10.1111/j.1748-1716.2009.01975.x 19245651

[B98] McCarron P. Smith G. D. Okasha M. McEwen J. (2000). Blood pressure in young adulthood and mortality from cardiovascular disease. Lancet 355 (9213), 1430–1431. 10.1016/s0140-6736(00)02146-2 10791531

[B99] McMurray R. G. Forsythe W. A. Mar M. H. Hardy C. J. (1987). Exercise intensity-related responses of beta-endorphin and catecholamines. Med. Sci. Sports Exerc 19 (6), 570–574. 10.1249/00005768-198712000-00005 2963188

[B100] Menz V. Marterer N. Amin S. B. Faulhaber M. Hansen A. B. Lawley J. S. (2019). Functional vs. Running low-volume high-intensity interval training: Effects on VO(2)max and muscular endurance. J. Sports Sci. Med. 18 (3), 497–504. 31427872 PMC6683610

[B101] Metcalfe R. S. Koumanov F. Ruffino J. S. Stokes K. A. Holman G. D. Thompson D. (2015). Physiological and molecular responses to an acute bout of reduced-exertion high-intensity interval training (REHIT). Eur. J. Appl. Physiol. 115 (11), 2321–2334. 10.1007/s00421-015-3217-6 26156806

[B102] Milanović Z. Sporiš G. Weston M. (2015). Effectiveness of high-intensity interval training (hit) and continuous endurance training for VO2max improvements: A systematic review and meta-analysis of controlled trials. Sports Med. 45 (10), 1469–1481. 10.1007/s40279-015-0365-0 26243014

[B103] Moldoveanu A. I. Shephard R. J. Shek P. N. (2001). The cytokine response to physical activity and training. Sports Med. 31 (2), 115–144. 10.2165/00007256-200131020-00004 11227979 PMC7101891

[B104] Moreno-Gómez C. Romaguera-Bosch D. Tauler-Riera P. Bennasar-Veny M. Pericas-Beltran J. Martinez-Andreu S. (2012). Clustering of lifestyle factors in Spanish University students: The relationship between smoking, alcohol consumption, physical activity and diet quality. Public Health Nutr. 15 (11), 2131–2139. 10.1017/s1368980012000080 22314203 PMC10271441

[B105] Murawska-Cialowicz E. Wolanski P. Zuwala-Jagiello J. Feito Y. Petr M. Kokstejn J. (2020). Effect of HIIT with Tabata protocol on serum irisin, physical performance, and body composition in men. Int. J. Environ. Res. Public Health 17 (10), 3589. 10.3390/ijerph17103589 32443802 PMC7277607

[B106] Nordestgaard B. G. (2017). A test in context: Lipid profile, fasting versus nonfasting. J. Am. Coll. Cardiol. 70 (13), 1637–1646. 10.1016/j.jacc.2017.08.006 28935041

[B107] Nunes P. R. P. Silva T. Carneiro M. A. S. Martins F. M. Souza A. P. Orsatti F. L. (2022). Functional high-intensity interval training is not equivalent when compared to combined training for blood pressure improvements in postmenopausal women: A randomized controlled trial. Clin. Exp. Hypertens. 44 (2), 127–133. 10.1080/10641963.2021.2001481 34749549

[B108] Nybo L. Sundstrup E. Jakobsen M. D. Mohr M. Hornstrup T. Simonsen L. (2010). High-intensity training versus traditional exercise interventions for promoting health. Med. Sci. Sports Exerc 42 (10), 1951–1958. 10.1249/MSS.0b013e3181d99203 20195181

[B109] Oliveira F. L. Patin R. V. Escrivão M. A. (2010). Atherosclerosis prevention and treatment in children and adolescents. Expert Rev. Cardiovasc Ther. 8 (4), 513–528. 10.1586/erc.09.170 20397826

[B110] Parolin M. L. Chesley A. Matsos M. P. Spriet L. L. Jones N. L. Heigenhauser G. J. (1999). Regulation of skeletal muscle glycogen phosphorylase and PDH during maximal intermittent exercise. Am. J. Physiol. 277 (5), E890–E900. 10.1152/ajpendo.1999.277.5.E890 10567017

[B111] Pearson R. C. Olenick A. A. Green E. S. Jenkins N. T. (2020). Tabata-style functional exercise increases resting and postprandial fat oxidation but does not reduce triglyceride concentrations. Exp. Physiol. 105 (3), 468–476. 10.1113/ep088330 31916294

[B112] Pescatello L. S. Franklin B. A. Fagard R. Farquhar W. B. Kelley G. A. Ray C. A. (2004). American College of Sports Medicine position stand. Exercise and hypertension. Med. Sci. Sports Exerc 36 (3), 533–553. 10.1249/01.mss.0000115224.88514.3a 15076798

[B113] Plotnikoff R. C. Costigan S. A. Williams R. L. Hutchesson M. J. Kennedy S. G. Robards S. L. (2015). Effectiveness of interventions targeting physical activity, nutrition and healthy weight for University and college students: A systematic review and meta-analysis. Int. J. Behav. Nutr. Phys. Act. 12, 45. 10.1186/s12966-015-0203-7 25890337 PMC4393577

[B114] Popowczak M. Rokita A. Koźlenia D. Domaradzki J. (2022). The high-intensity interval training introduced in physical education lessons decrease systole in high blood pressure adolescents. Sci. Rep. 12 (1), 1974. 10.1038/s41598-022-06017-w 35132123 PMC8821617

[B115] Rankinen T. Church T. S. Rice T. Bouchard C. Blair S. N. (2007). Cardiorespiratory fitness, BMI, and risk of hypertension: The HYPGENE study. Med. Sci. Sports Exerc 39 (10), 1687–1692. 10.1249/mss.0b013e31812e527f 17909393

[B116] Regitz-Zagrosek V. Lehmkuhl E. Mahmoodzadeh S. (2007). Gender aspects of the role of the metabolic syndrome as a risk factor for cardiovascular disease. Gend. Med. 4, S162–S177. 10.1016/s1550-8579(07)80056-8 18156101

[B117] Ridker P. M. Everett B. M. Thuren T. MacFadyen J. G. Chang W. H. Ballantyne C. (2017). Antiinflammatory therapy with canakinumab for atherosclerotic disease. N. Engl. J. Med. 377 (12), 1119–1131. 10.1056/NEJMoa1707914 28845751

[B118] Riediger N. D. Clara I. (2011). Prevalence of metabolic syndrome in the Canadian adult population. Cmaj 183 (15), E1127–E1134. 10.1503/cmaj.110070 21911558 PMC3193129

[B119] Rosenblat M. A. Granata C. Thomas S. G. (2022). Effect of interval training on the factors influencing maximal oxygen consumption: A systematic review and meta-analysis. Sports Med. 52 (6), 1329–1352. 10.1007/s40279-021-01624-5 35041180

[B120] Ross R. Dagnone D. Jones P. J. Smith H. Paddags A. Hudson R. (2000). Reduction in obesity and related comorbid conditions after diet-induced weight loss or exercise-induced weight loss in men. A randomized, controlled trial. Ann. Intern Med. 133 (2), 92–103. 10.7326/0003-4819-133-2-200007180-00008 10896648

[B121] Ryan A. S. Li G. McMillin S. Prior S. J. Blumenthal J. B. Mastella L. (2021). Pathways in skeletal muscle: Protein signaling and insulin sensitivity after exercise training and weight loss interventions in middle-aged and older adults. Cells 10 (12), 3490. 10.3390/cells10123490 34943997 PMC8700073

[B122] Ryan B. J. Schleh M. W. Ahn C. Ludzki A. C. Gillen J. B. Varshney P. (2020). Moderate-intensity exercise and high-intensity interval training affect insulin sensitivity similarly in obese adults. J. Clin. Endocrinol. Metab. 105 (8), e2941–e2959. 10.1210/clinem/dgaa345 32492705 PMC7347288

[B123] Sabbahi A. Arena R. Elokda A. Phillips S. A. (2016). Exercise and hypertension: Uncovering the mechanisms of vascular control. Prog. Cardiovasc Dis. 59 (3), 226–234. 10.1016/j.pcad.2016.09.006 27697533

[B124] Saeed A. Kampangkaew J. Nambi V. (2017). Prevention of cardiovascular disease in women. Methodist Debakey Cardiovasc J. 13 (4), 185–192. 10.14797/mdcj-13-4-185 29744010 PMC5935277

[B125] Sallam N. Laher I. (2016). Exercise modulates oxidative stress and inflammation in aging and cardiovascular diseases. Oxid. Med. Cell Longev. 2016, 7239639. 10.1155/2016/7239639 26823952 PMC4707375

[B126] Sawyer B. J. Tucker W. J. Bhammar D. M. Ryder J. R. Sweazea K. L. Gaesser G. A. (2016). Effects of high-intensity interval training and moderate-intensity continuous training on endothelial function and cardiometabolic risk markers in obese adults. J. Appl. Physiol. 121 (1), 279–288. 10.1152/japplphysiol.00024.2016 27255523 PMC4967258

[B127] Scarapicchia T. M. Sabiston C. M. Faulkner G. (2015). Exploring the prevalence and correlates of meeting health behaviour guidelines among University students. Can. J. Public Health 106 (3), e109–e114. 10.17269/cjph.106.4784 26125235 PMC6972311

[B128] Scott S. P. Mj D. E. S. Koehler K. Petkus D. L. Murray-Kolb L. E. (2016). Cardiorespiratory fitness is associated with better executive function in young women. Med. Sci. Sports Exerc 48 (10), 1994–2002. 10.1249/mss.0000000000000974 27183121

[B129] Sharp P. Caperchione C. (2016). The effects of a pedometer-based intervention on first-year University students: A randomized control trial. J. Am. Coll. Health 64 (8), 630–638. 10.1080/07448481.2016.1217538 27471879

[B130] Silva M. N. Markland D. Carraça E. V. Vieira P. N. Coutinho S. R. Minderico C. S. (2011). Exercise autonomous motivation predicts 3-yr weight loss in women. Med. Sci. Sports Exerc 43 (4), 728–737. 10.1249/MSS.0b013e3181f3818f 20689448

[B131] Skovgaard E. L. Obling K. Maindal H. T. Rasmussen C. Overgaard K. (2019). Unprompted vigorous physical activity is associated with higher levels of subsequent sedentary behaviour in participants with low cardiorespiratory fitness: A cross-sectional study. Eur. J. Sport Sci. 19 (7), 1004–1013. 10.1080/17461391.2019.1574905 30758264

[B162] Smith-Jackson T. Reel J.J. (2012). Freshmen women and the “Freshman 15”: perspectives on prevalence and causes of college weight gain. J Am Coll Health. 60 (1), 14–20. 10.1080/07448481.2011.555931 22171725

[B132] Son W. M. Sung K. D. Cho J. M. Park S. Y. (2017). Combined exercise reduces arterial stiffness, blood pressure, and blood markers for cardiovascular risk in postmenopausal women with hypertension. Menopause 24 (3), 262–268. 10.1097/gme.0000000000000765 27779565

[B133] Soriano-Ayala E. Amutio A. Franco C. Mañas I. (2020). Promoting a healthy lifestyle through mindfulness in university students: A randomized controlled trial. Nutrients 12 (8), 2450. 10.3390/nu12082450 32824061 PMC7468720

[B134] Støren Ø. Helgerud J. Sæbø M. Støa E. M. Bratland-Sanda S. Unhjem R. J. (2017). The effect of age on the V˙O2max response to high-intensity interval training. Med. Sci. Sports Exerc 49 (1), 78–85. 10.1249/mss.0000000000001070 27501361

[B135] Sultana R. N. Sabag A. Keating S. E. Johnson N. A. (2019). The effect of low-volume high-intensity interval training on body composition and cardiorespiratory fitness: A systematic review and meta-analysis. Sports Med. 49 (11), 1687–1721. 10.1007/s40279-019-01167-w 31401727

[B136] Sun S. Zhang H. Kong Z. Shi Q. Tong T. K. Nie J. (2019). Twelve weeks of low volume sprint interval training improves cardio-metabolic health outcomes in overweight females. J. Sports Sci. 37 (11), 1257–1264. 10.1080/02640414.2018.1554615 30563431

[B137] Sweeney M. M. (2011). Initiating and strengthening: College and university instructional physical activity programs. J. Phys. Educ. Recreat. dance 82 (4), 17–21. 10.1080/07303084.2011.10598609

[B138] Tabata I. Nishimura K. Kouzaki M. Hirai Y. Ogita F. Miyachi M. (1996). Effects of moderate-intensity endurance and high-intensity intermittent training on anaerobic capacity and VO2max. Med. Sci. Sports Exerc 28 (10), 1327–1330. 10.1097/00005768-199610000-00018 8897392

[B139] Tabata I. (2019). Tabata training: One of the most energetically effective high-intensity intermittent training methods. J. Physiol. Sci. 69 (4), 559–572. 10.1007/s12576-019-00676-7 31004287 PMC10717222

[B140] Tcymbal A. Andreasyan D. Whiting S. Mikkelsen B. Rakovac I. Breda J. (2020). Prevalence of physical inactivity and sedentary behavior among adults in Armenia. Front. Public Health 8, 157. 10.3389/fpubh.2020.00157 32432072 PMC7214797

[B141] Tjønna A. E. Lee S. J. Rognmo Ø. Stølen T. O. Bye A. Haram P. M. (2008). Aerobic interval training versus continuous moderate exercise as a treatment for the metabolic syndrome: A pilot study. Circulation 118 (4), 346–354. 10.1161/circulationaha.108.772822 18606913 PMC2777731

[B142] Trapp E. G. Chisholm D. J. Freund J. Boutcher S. H. (2008). The effects of high-intensity intermittent exercise training on fat loss and fasting insulin levels of young women. Int. J. Obes. (Lond) 32 (4), 684–691. 10.1038/sj.ijo.0803781 18197184

[B143] Vainshelboim B. Brennan G. M. LoRusso S. Fitzgerald P. Wisniewski K. S. (2019). Sedentary behavior and physiological health determinants in male and female college students. Physiol. Behav. 204, 277–282. 10.1016/j.physbeh.2019.02.041 30831185

[B144] Vella-Zarb R. A. Elgar F. J. (2009). The 'freshman 5': A meta-analysis of weight gain in the freshman year of college. J. Am. Coll. Health 58 (2), 161–166. 10.1080/07448480903221392 19892653

[B145] Venables M. C. Achten J. Jeukendrup A. E. (2005). Determinants of fat oxidation during exercise in healthy men and women: A cross-sectional study. J. Appl. Physiol. 98 (1), 160–167. 10.1152/japplphysiol.00662.2003 15333616

[B146] Viana R. B. de Lira C. A. B. Naves J. P. A. Coswig V. S. Del Vecchio F. B. Gentil P. (2019). Tabata protocol: A review of its application, variations and outcomes. Clin. Physiol. Funct. Imaging 39 (1), 1–8. 10.1111/cpf.12513 29608238

[B147] von Bothmer M. I. Fridlund B. (2005). Gender differences in health habits and in motivation for a healthy lifestyle among Swedish University students. Nurs. Health Sci. 7 (2), 107–118. 10.1111/j.1442-2018.2005.00227.x 15877687

[B148] Wen D. Utesch T. Wu J. Robertson S. Liu J. Hu G. (2019). Effects of different protocols of high intensity interval training for VO(2)max improvements in adults: A meta-analysis of randomised controlled trials. J. Sci. Med. Sport 22 (8), 941–947. 10.1016/j.jsams.2019.01.013 30733142

[B149] Whatnall M. C. Patterson A. J. Chiu S. Oldmeadow C. Hutchesson M. J. (2019). Feasibility and preliminary efficacy of the eating advice to students (eats) brief web-based nutrition intervention for young adult university students: A pilot randomized controlled trial. Nutrients 11 (4), 905. 10.3390/nu11040905 31018565 PMC6520699

[B150] Wu S. Fisher-Hoch S. P. Reininger B. McCormick J. B. (2016). Recommended levels of physical activity are associated with reduced risk of the metabolic syndrome in Mexican-Americans. PLoS One 11 (4), e0152896. 10.1371/journal.pone.0152896 27054324 PMC4824434

[B151] Yang L. Yan J. Tang X. Xu X. Yu W. Wu H. (2016). Prevalence, awareness, treatment, control and risk factors associated with hypertension among adults in southern China, 2013. PLoS One 11 (1), e0146181. 10.1371/journal.pone.0146181 26784948 PMC4718602

[B152] Yang Yx W. G. Pan X. C. (2002). China food composition Tables 2002. Beijing, China: Beijing University Medical Press.

[B153] Yano Y. (2021). Blood pressure in young adults and cardiovascular disease later in life. Am. J. Hypertens. 34 (3), 250–257. 10.1093/ajh/hpab005 33821946

[B154] Zhang H. Tong T. K. Kong Z. Shi Q. Liu Y. Nie J. (2021). Exercise training-induced visceral fat loss in obese women: The role of training intensity and modality. Scand. J. Med. Sci. Sports 31 (1), 30–43. 10.1111/sms.13803 32789898

[B155] Zhang H. Tong T. K. Qiu W. Zhang X. Zhou S. Liu Y. (2017). Comparable effects of high-intensity interval training and prolonged continuous exercise training on abdominal visceral fat reduction in obese young women. J. Diabetes Res. 2017, 5071740. 10.1155/2017/5071740 28116314 PMC5237463

[B156] Zhao W. Hasegawa K. Chen J. (2002). The use of food-frequency questionnaires for various purposes in China. Public Health Nutr. 5 (6), 829–833. 10.1079/phn2002374 12638592

[B157] Zhou B. F., (2002). Predictive values of body mass index and waist circumference for risk factors of certain related diseases in Chinese adults-study on optimal cut-off points of body mass index and waist circumference in Chinese adults. Biomed. Environ. Sci. 15 (1), 83–96. 12046553

[B158] Zhu Y. Xian X. Wang Z. Bi Y. Chen Q. Han X. (2018). Research progress on the relationship between atherosclerosis and inflammation. Biomolecules 8 (3), 80. 10.3390/biom8030080 30142970 PMC6163673

[B159] Zouhal H. Jacob C. Delamarche P. Gratas-Delamarche A. (2008). Catecholamines and the effects of exercise, training and gender. Sports Med. 38 (5), 401–423. 10.2165/00007256-200838050-00004 18416594

